# Revised taxon definition in European *Cortinarius* subgenus *Dermocybe* based on phylogeny, chemotaxonomy, and morphology

**DOI:** 10.1007/s11557-024-01959-z

**Published:** 2024-04-05

**Authors:** Lesley Rosina Huymann, Anna Hannecker, Turrini Giovanni, Kare Liimatainen, Tuula Niskanen, Maraike Probst, Ursula Peintner, Bianka Siewert

**Affiliations:** 1 Department of Microbiology, University Innsbruck, Technikerstraße 25d, 6020 Innsbruck, Austria; 2 Department of Pharmacognosy, Institute of Pharmacy, Center for Chemistry and Biomedicine, University Innsbruck, Innrain 80 - 82/IV, 6020 Innsbruck, Austria; 3 Jodrell Laboratory, Royal Botanic Gardens, Kew, Surrey TW9 3AB UK; 4 Botany Unit, Finnish Museum of Natural History, University of Helsinki, P.O. Box 7, 00014 Helsinki, Finland; 5 AMB Bresadola – Sezione Bolzano, Bolzano, Italy

**Keywords:** Anthraquinone pigments, Integrative taxonomy, Identification key, Central alpine range, Coniferous forests

## Abstract

*Cortinarius* (Fr.) Fr. is one of the most species-rich genera in the *Agaricales* (*Basidiomycota*). *Cortinarius* subgen. *Dermocybe* (Fr.) Trog includes brightly coloured Cortinarii with anthraquinone pigments. The chemotaxonomic approach has always been as important as classical methods for species definition of *Dermocybe* and helped to improve overall species concepts. However, some species concepts within this group remain unclear. We therefore address this topic based on a combined phylogenetic, morphological, and pigment-chemical approach. For this, sequence data, HPLC–MS pigment profiles and spore sizes were included were included to obtain a better resolution of taxa. The study was based on 173 recent collections and 12 type specimens. A total of 117 rDNA ITS sequences were produced from the collections in this study, 102 sequences were retrieved from databases. We could detect and clearly delimit 19 *Dermocybe* species occurring in central European habitats, from which 16 are discussed in detail. Additionally, we grouped the detected anthraquinone pigments into four groups. This detailed analysis of dermocyboid *Cortinarius* species occurring in a restricted number of habitat types confirmed our hypothesis that species diversity is much higher than currently assumed. This high diversity is blurred by too wide and incorrect species concepts of several classical species like *C. croceus* and *C. cinnamomeus*. Molecular and chemotaxonomical studies carried out together with careful phenotypical analyses resulted in a good differentiation of species. A key is presented for these taxa to allow a better identification of *Cortinarius* subgenus *Dermocybe* spp. occurring in Central Europe mainly in the alpine range.

**Supplementary Information:**

The online version contains supplementary material available at 10.1007/s11557-024-01959-z.

## Introduction

Of all known macrofungi, *Cortinarius* (Fr.) Fr. (*Cortinariaceae*, *Agaricales*) is one of the genera with both the highest number of species and a high level of uncertainty concerning delimitation of taxa. The genus was recently split (Liimatainen et al. 2022), but the subgenus *Dermocybe* remains part of the genus *Cortinarius*. However, species delimitation is difficult in all its genera and groups (Liu et al. 1997; Stefani et al. 2014). The characters available for discrimination of *Cortinarius* taxa include molecular, morphological, and chemo-taxonomical properties (Arnold et al. 1987; Kõljalg et al. 2013) as well as ecological features such as habitat or associated hosts (Torbjørn and Høiland 1988). Developments in molecular biology and phylogenetical analysis enable incorporating DNA and evolutionary aspects into species hypotheses (Liu et al. 1997; Stefani et al. 2014). Due to those developments, many taxonomic concepts have been updated (Liu et al. 1995, 1997; Peintner et al. 2004; Niskanen et al. 2012; Stensrud et al. 2014).

The chemotaxonomic approach has always been an important method for the distinction of species within the subgenus *Dermocybe* (Fr.) Trog. This subgenus was initially delimited from other subgenera of *Cortinarius* based on the presence of anthraquinone pigments (with a few exceptions) (Gruber 1970; Høiland & Holst-Jensen 2000; Moser 1976). However, its status was repeatedly debated (Orton 1958; Gruber 1970; Moser 1978; Høiland 1983; Liu et al. 1997; Kuhnert-Finkernagel and Peintner 2003), and the earlier, broader morphology- and chemotaxonomy-based concept of *Dermocybe *sensu lato was revised into an evolutionary-based concept circumscribing only taxa within the phylogenetic lineage of *Dermocybe *sensu stricto.

*Dermocybe *sensu lato is in the following referred to as dermocyboid species. It consits of agaricoid, small to medium-sized, brightly coloured fungi (yellow, red, brownish to olive colours) with a dry stipe and a dry to slightly slimy, silky, or even waxy pileus; the pileipellis is somewhat duplex with a poorly developed hypoderm. The lamellae are adnate or adnexed and of yellow, red, orange or green colour; the veil is insignificant, and a cortina is present. The smell is often indistinct or of radish (Keller 1982; Moser 1972, 1976; Høiland 1983, 1985; Kidd et al. 1985; Liu et al. 1997; Knudsen 2008; Stefani et al. 2014; Soop 2021; Liimatainen et al. 2022).

The phylogenetically well supported subgenus *Dermocybe *sensu stricto circumscribes a Northern Hemisphere *Cortinarius* lineage which includes the type of the subgenus *C*. *cinnamomeus* (Stensrud et al. 2014). The colouration of those vibrantly coloured fruitbodies is caused entirely by anthraquinone pigments. The biological purpose thereof may be the prevention of insect grub (Moser 1972; Siewert 2021). Dermocyboid Cortinarii have a species-specific pigment composition (qualitatively and quantitatively) (Keller 1982; Siewert et al. 2022). The pigments can also be used for dying wool and other commodities (Knudsen and Vesterholt 2008).

*Dermocyboid* fungi and their pigments have been intensely studied in Europe, North-America, and Australia (Kögel and Postowsky 1925; Gruber 1970; Keller 1982; Moser 1976; Høiland 1983, 1985; Keller et al. 1988; Gill 1995; Liu et al. 1997; Niskanen et al. 2013; Soop 2016). There are two main approaches for working with dermocyboid species: several studies address the structure and function of pigments only (Kögel and Postowsky 1925; Steglich and Austel 1966; Steglich et al. 1969a, b; Siewert 2021; Hammerle et al. 2022). Other studies tried to combine the pigment profiles with other properties, in order to gain a more comprehensive insight into the relationship and evolutionary history of these fungi (Gruber 2009; Keller 1982; Keller et al. 1988; Kidd et al. 1985). The latter can be adressed as a combination of chemotaxonomy with phylogenetic systematics.

Chemotaxonomic classification within the subgenus started as early as 1925, after successful isolation of emodin and dermocybin from *Cortinarius sanguineus (*Wulf.) Gray (Kögel and Postowsky 1925; Steglich and Austel 1966; Steglich et al. 1969a, b). Thereafter, other anthraquinone-pigments, like dermolutein, dermorubin, physcion, and dermoglaucin, were isolated from the red-pigmented species *C. sanguineus* and *C. semisanguineus* (Fr.) Gillet (Steglich and Austel 1966; Steglich et al. 1969a, b; Steglich et al. 1982).

There is a typical anthraquinone pigment composition for all dermocyboid species, and the individual pigment profiles can be considered as reliable characters for identifying dermocyboid species (Høiland 1983; Arnold et al. 1987). The methods applied for obtaining these pigment profiles range from paper and thin layer chromatography (TLC) (Gruber 1970; Keller 1982) to high pressure liquid chromatography (HPLC) combined with a diode array detector (DAD) and/or mass spectrometer (MS). The best resolution can be obtained with HPLC due to its high sensitivity and reproducibility (Fiala et al. 2021).

Pigments were grouped into different pigment types by Keller (1982) and since then, this system has been widely adopted in an amplified and optimized way (Gruber 1970; Keller 1982; Høiland 1983; Arnold et al. 1987; Keller et al. 1988; Liu et al. 1995). Over time, the taxonomic groups formed based on the pigments were much discussed and there are different concepts for the best taxon-grouping founded on pigment types. Høiland (1983) wrote that pigment types classify the Northern Hemisphere dermocyboid *Cortinarii* into the three pigment groups: Dermocybe, Malicoriae, and Sanguineae. A forth group, namely Olivaceofuscus, can be found in both the Northern and Southern Hemisphere (Høiland 1983). The overlap between these pigment groups and dermocyboid *Cortinarius* sections is yet to be confirmed. The pigment group Dermocybe circumscribes species with yellow to orange lamellae. The pigment group Malicoriae circumscribes specimens with deep orange lamellae. The pigment group Sanguineae circumscribes species with red pigments, but it is not clear if this group splits into two clades, one around *C. sanguineus,* and the other around *C. semisanguineus* (Keller 1982). The Olivaceofuscus group has mainly yellow to olive fruit bodies.

Currently, DNA-based phylogeny and phylogenomics appear to be one of the best tools to resolve species complexes (Stefani et al. 2014). A majority of the studies about *Cortinarius* have used the rDNA ITS region for species delimitation, but in a few cases it failed in previous studies to discriminate closely related species due to low sequence divergence (Garnica et al. 2005). Therefore, using integrated taxonomy: combining ITS and other characters as pigment characteristics, morphology and ecology, are useful to be included into a solid species delimitation of dermocyboid Cortinarii, specially in cases of closely related sister species for which ITS alone does not provide a clear distinction in the phylogeny. Like all other *Cortinarius* spp., dermocyboid species are mycorrhizal fungi (Knudsen 2008), therefore, cultivation is not easily possible and *in-vitro* experiments, e.g. mating studies, are not an option.

Assigning correct names for the species delimited is often problematic. Part of the species are not yet described and it is widely known that databases include many wrongly assigned sequences and lack data from type specimens (Nilsson et al. 2006). Hence, BLAST results often lead to a wrong determination. Furthermore, there are valid—enigmatic, forgotten, or neglected—species which are waiting for re-discovery based on type studies. For example, *C. holoxanthus*, or *C. ominosus* which are often identified as the commonly wrong used epithets *C. croceus* or *C. semisanguineus*, respectively. Hence, sequencing of type specimens is prerequisite to enable correct naming of species through BLAST.

Once the species have been delimited based on integrated taxonomy or there is no DNA or pigment-based data available, morphological characteristics most useful for identification of the species can be selected. The most important morphological traits used are the colouration of stipe, pileus and lamellae as well as the structure of the pileipellis, the characteristics of the spores (size, form, and ornamentation), and the habitus (Keller 1982; Høiland 1983). The advantages of a morphological determination are that it is fast and requires a minimum of equipment. Nevertheless, it is very difficult, requires experience and also with that often leads to wrongly assigned species. Furthermore, one would need literature, which is presently hindered by the fact that, so far, identification keys do not include all known species of a habitat. Therefore, we want to present an updated key, especially for coniferous forest habitats of the Alps, on which we mainly focussed here.

The aim of this study was to delimit and redefine dermocyboid Cortinarii based on a blurry set of available characters*.* As a start, we restricted our range of investigation to species occurring in the Central European alpine environment. We also wanted to test and compare the results obtained by three different approaches – morphology, phylogeny, and pigment chemistry – in order to define reliable characters for species differentiation. By integrating sequences generated from type material into the study, we could also address the correct naming of species. We circumscribe 15 species, and with the obtained results, we can confirm or reject species widely applied epithets, detect synonyms, and re-discover forgotten species. We provide an overview of the most abundant dermocyboid *Cortinarii* found in coniferous forests of the alpine environment, and present an identification key as a basic tool for a fast and easy species identification in this environment. This should form a solid base for future studies circumscribing dermocyboid Cortinarii in a wider ecological context.

## Methods

### Specimen sampling

In this study, 161 collections of *Cortinarius* fruit bodies were gathered and 12 samples from type material were included, thus allowing for unambiguous delimitation of these taxa. The list of the collections of species examined in this study are provided (See Additional Material Table 2). The material was collected in samples of around 5 to 10 specimens, apart from a few smaller samples, and includes species of the subgenus *Dermocybe* used in our earlier studies (Siewert et al. 2022; Hannecker et al. 2023). Material was collected in Central Europe, mostly in coniferous alpine habitat. Voucher specimens are deposited in the Herbarium der Tiroler Landesmuseen Ferdinandeum (IBF) (Krajnc-Straße 1, 6060 Hall, Austria).

### Morphological studies

Most of the collections were photographed in a fresh state. Morphological descriptions were made based on fresh material. Macro chemical reactions were carried out with KOH 30%. Possible fluorescence under UV was observed at a wavelength of 250 nm and 350 nm based on dry material. Light microscopy was carried out with a Nikon Eclipse 600 and either with fresh material or with dried material soaked in water or KOH 3% before visualization. Measurements were carried out using the imaging software Nis-Elements D (© 2021 Nikon Europe B.V.; URL https://www.microscope.healthcare.nikon.com/de_EU/products/software/niselements/niselements-documentation). Spore measurements were carried out in KOH 3% under a 100 × oil immersions objective, based on ripe spores taken from the cortina as far as possible. For every species, 30 spores were measured in order to allow for statistical evaluation of spore size. The results are presented in the following scheme: (min) MV ± *sd* (max) x (min) MV ± *sd* (max) (n = x). The length/width ratio Q was calculated and a 95% confidence interval was applied for the scatterplot.

### DNA extraction, PCR amplification and sequencing

DNA was extracted from dried or fresh fruitbody material with the CTAB-Method (Peintner et al. 2001). Usually a small piece (few milligrams) of lamella was taken. For DNA extraction, chemicals from Merck (Merck KGaA, Darmstadt, Germany) were used. For PCR amplification of the nuclear ribosomal RNA ITS gene (Siewert et al. 2022), the primer pair ITS1 and ITS4 was used. For a few problematic samples, the following primer pairs were used: ITS1 and ITS2 to gain the ITS1 region, and ITS3 and ITS4 to gain the ITS2 region. The primers were produced by Microsynth (Microsynth AG, Balgach, Switzerland). Other PCR reagents were from Procomcure *Biotech* (Procomcure Biotech Thalgau, Austria). For sequencing, the PCR products were sent to Microsynth AG (Schützenstrasse 15 P.O. Box 9436, Balgach, Switzerland).

### Phylogenetic analyses

The program Sequencher 5.2.3 (Gen Codes Cooperation, http://www.genecodes.com/) was used for editing and assembling of sequences. The 112 generated sequences were deposited in GenBank (URL https://www.ncbi.nlm.nih.gov/GenBank/) (See Additional Material Table 2). Blast searches were carried out in the databases GenBank and UNITE (https://unite.ut.ee/), and 96 closely related sequences were downloaded and used for phylogenetic analysis. All sequences were aligned and analysed in MEGA X (MEGA software, URL https://www.megasoftware.net/). The maximum likelihood (ML) tree was produced using the Hasegawa-Kishino-Yano model (Hasegawa et al. 1985) with discrete Gamma distribution (G). The tree with the highest log likelihood is shown (-3320.34). All sites were used. Maximum parsimony (MP) with 500 replications was used for calculating bootstrap values (Kumar et al. 2018). A Baysian inference tree was calculated with MrBayes 3.2.7a (MrBayes: Bayesian Inference of Phylogeny, URL http://nbisweden.github.io/MrBayes/index.html) (Huelsenbeck 2001). Here, a Markov Chain Monte Carlo (MCMC) analysis was performed with the following setting: a gamma shape parameter, four runs with 5 M generations, sampling every 1000 generations, and the first 25% of the samples were discarded as burn-infraction before the statistics were calculated. *Cortinarius cinnabarinus* and *C*. *anthracinus* were used as outgroup. They are morphologically similar but do not belong to subgenus *Dermocybe* (Høiland 1983; Høiland and Holst-Jensen 2000) or the Dermocybe lineage (Liimatainen et al. 2022).

### Chromatography and identification of pigments

For the identification of the pigments, a previously established HPLC–DAD-(MS) method was used (Siewert et al. 2022). Solvents used for pigment-extraction were purchased from VWR (VWR International, Vienna, Austria), whereby acetone was additionally distilled prior to use. For HPLC experiments, solvents from Merck (Merck KGaA, Darmstadt, Germany) of pro analysis (p.a.) quality, were used. Ultrapure water was generated using the Sartorius arium® 611 UV purification system (Sartorius AG, Göttingen, Germany). The peaks of the chromatograms were assigned to the pigments based on their fragmentation pattern, UV–Vis absorption pattern, and their retention time. Peak threshold analysis was set to 5%; when needed, peaks were added manually for description. For peak picking as well as chromatogram visualization, Origin Pro 2020 was used (Origin Lab Cooperation, URL https://www.originlab.com/2020, USA). To cover all colour ranges, the chromatograms recorded at 428, 478, and 519 nm were added together. Trace and main pigments were defined relative to the highest peak (set to 100%) in each chromatogram (i.e., trace pigment < 10% < main pigment).

### Correlation between pigment type and phylogeny

A tanglegram was calculated using R (Version 4.0.2) in order to correlate the pigment profiles and the phylogeny. For analysis, packages vegan (Oksanen et al. 2017), biotools (da Silva et al. 2017), and dendextend (Galili 2015) were used. For the pigment profiles, the absolute pigment quantity relative to the highest peak in each chromatogram was calculated. Based on this matrix, a pairwise Bray Curtis distance matrix was calculated. For the phylogeny, a similar Bray Curtis distance matrix was created from the ITS based alignment. Based on those distance matrices, dendrograms (Ward.D) were created via hierarchical clusters, which were then compared. The correlation between the distance matrices was calculated by Mantel-test (999 permutations).

### Statistical methods and graphic programms

All statistical analyses were performed in R Version 4.0.2 (2020–06-22) (R Foundation for Statistical Computing, Vienna, Austria; URL https://www.R-project.org/) (Team R.D.C. 2009). For comparing of Q-values and spore sizes, a Bonferroni t-test was applied (package psych) (Revelle 2023). For the scatterplot of the spore sizes, the package ggplot2 (Wickham 2016) was used. For creating the artwork, Inkscape 1.3 (URL https://inkscape.org/de/) was used.

### Equipment

A Sartorius Cubis^®^-series balance (Sartorius AG, Göttingen, Germany) and a Kern 440 balance (KERN & SOHN GmbH, Balingen-Frommern, Germany) was used, as well as the ultrasonic baths Sonorex RK 52 and Sonorex RK 106 (BANDELIN electronic GmbH & Co. KG, Berlin, Germany). Mixing was performed with the vortex mixer Vortex-Genie 2 (Scientific Industries, Inc., Bohemia, New York, USA). Incubation and mixing required for DNA extraction was done with an Eppendorf Thermomixer comfort (Eppendorf AG, Germany, Hamburg). Centrifugation was done with the Eppendorf centrifuge 5804 R (Eppendorf AG, Germany) and the Eppendorf centrifuge 5415 R (Eppendorf AG, Germany). HPLC–DAD-ESI–MS analysis was carried out with the modular system Agilent Technologies 1260 Infinity II equipped with a quaternary pump, vial sampler, column thermostat, diode-array detector, and an ion trap mass spectrometer (amaZon, Bruker, Bremen, Germany). Moreover, the HPLC-system SHIMADZU HPLC/UPLC-UFLC XR, with binary pump, vial sampler, column thermostat, and diode-array detector was used (SHIMADZU CORPORATION, Kyoto, Japan). For PCR the Theromcycler Peqlab Primus 96 advanced (Peqlab Biotechnologie GmbH, Erlangen, Germany). For visualization of PCR-products, the electrophorese chamber RunOne Casting System (Embi Tec, San Diego, California, USA) and the Bio Rad Gel Doc EZ Imager 1708270 together with the Bio Rad Blue sample tray were used (Bio-Rad Laboratories, Hercules, California, USA).

### Identification key

For constructing a dichotomous identification key, the morphological characters noted during this study were used as well as the following literature: (Moser 1976, 1978; Knudsen 2008; Niskanen et al. 2012; Niskanen 2014; Soop 2021).

## Results

### Phylogenetic species recognition

**Fig. 1:**
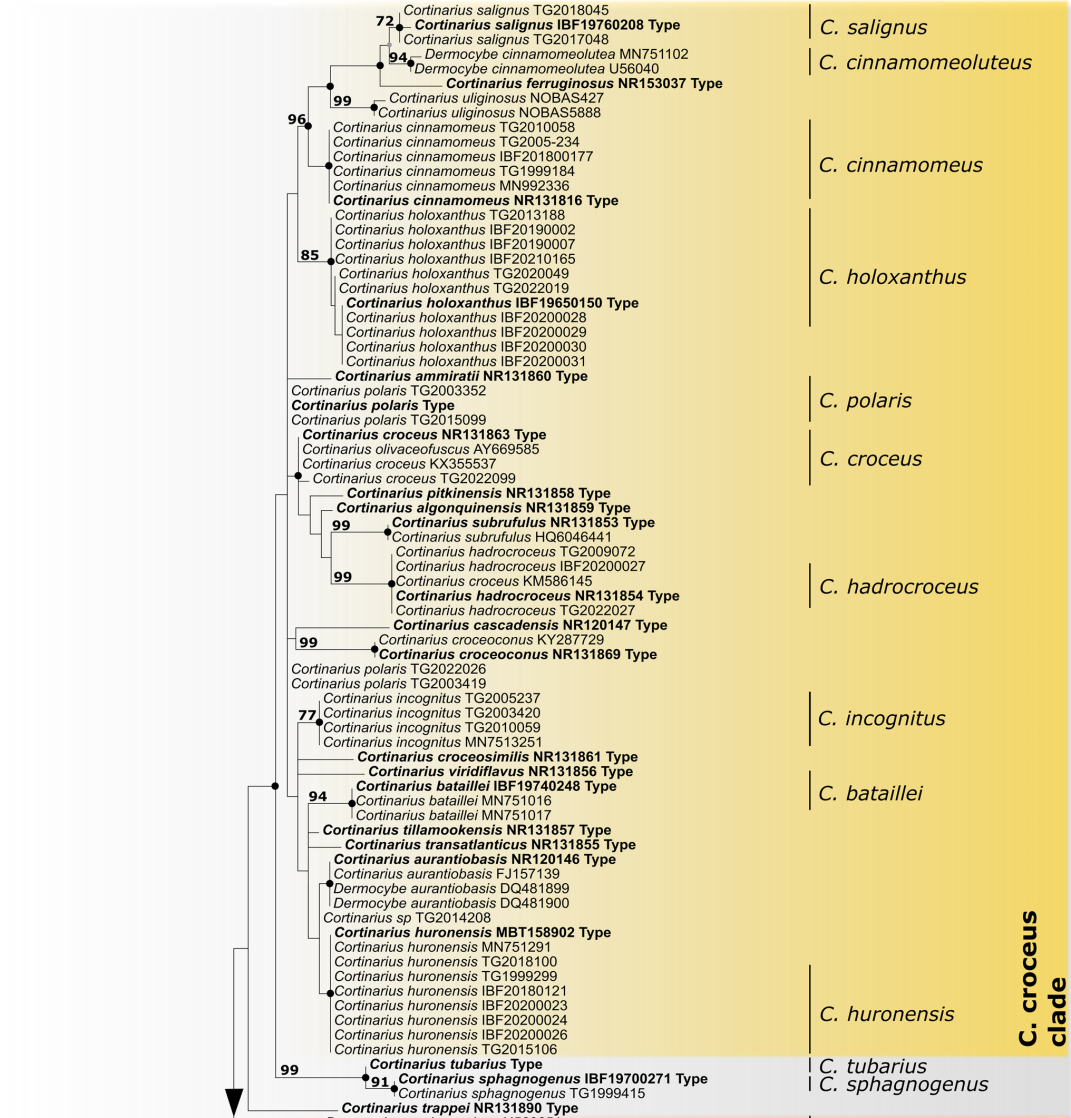

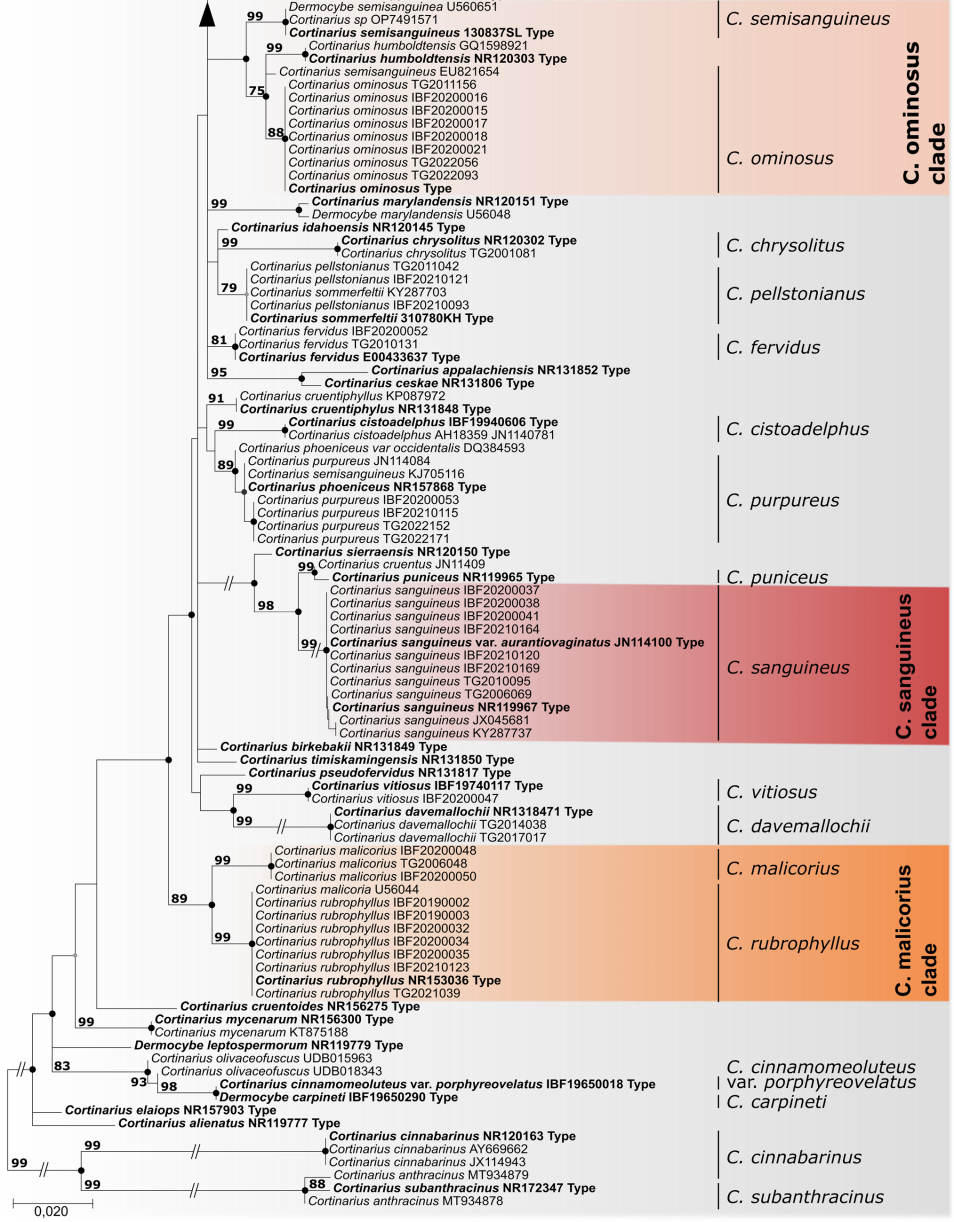
Phylogenetic relationship of European dermocyboid *Cortinarius* species based on a maximum likelihood tree. Maximum parsimony bootstrap support values > 80% are given on the branches; black and small grey dots on the branches represent Bayesian posterior probabilities > 0.96 and > 0.80, respectively; GenBank or voucher numbers are given behind the species’ names; type sequences are printed in bold

In this study, a total of 112 new rDNA ITS sequences were generated, including 12 sequences from newly sequenced type material. Dermocyboid Cortinarii fall in a well-supported phylogenetic lineage (BPP 1, BS 99%) which is clearly distinct from the outgroup (*C. cinnabarinus*, *C. anthracinus, C. subanthracinus*) (Fig. 1). Given sufficient sampling size, species are usually well-resolved. The phylogenetic analysis revealed that the diversity of dermocyboid *Cortinarius* spp. is generally very high with 32 European species intermixed with at least 25 species from North America or the Southern Hemisphere. The exact number of taxa is not clear since from part of them i.e. those only differing by some bases and indels from the known species and only represented by single collection, more materials would be needed for a reliable species delimitation. This leads to frequent misidentification of deposited sequences potentially representing new species. In the following, only European taxa will be discussed. Taxa from America or the Southern Hemisphere were included in the analysis for a better delimitation of species. For a list of all investigated collections and information concerning their voucher numbers, GenBank numbers, and origin see Additional Material Table 2.

The dermocyboid *Cortinarius* diversity is comparatively high in the alpine environment, our samples represent at least 19 species. Our collecions of core subgenus *Dermocybe* fell into the following four well defined clades which can partly be addressed by their predominant colour of lamellae: i) The “yellow” lineage or the C. croceus clade includes the European taxa *C. bataillei, C. cinnamomeoluteus, C*. *cinnamomeus, C. croceus, C. ferruginosus, C. hadrocroceus, C. holoxanthus, C. polaris, C. salignus, C. sphagnogenu*s, *C. tubarius, C. uliginosus,* and the North American taxa *C. ammiratii*, *C*. *algonquinensis*, *C. aurantiobasis, C*. *croceosimilis*, *C. huronensis, C. incognitus C*. *pitkiniensis*, *C*. *subrufulus*, *C*. *tillamookensis*, *C. transatlanticus*, *C. viridiflavus*. ii) The “red” C. semisanguineus clade includes our samples of *C. ominosus,* and reference sequences from *C. semisanguineus* and *C. tinctorum*, as well as *C. humboldtensis* from North America*.* iii) The “all-over red” C. sanguineus clade includes our samples of *C. sanguineus* var. *aurantiovaginatus, C. sanguineus* and *C. puniceus, C. cruentus,* as well as *C. sierraensis* from North America. iv) The “orange” C. malicorius clade includes *C*. *malicorius* and *C*. *rubrophyllus*. The following species within the Dermocybe clade did not fall into subclades, but sister-group relationships were resolved for *C. pellstonianus, C*. *fervidus*, *C*. *vitiosus*, *C. purpureus* and *C. cistoadelphu*s.

### Pigment profiling

**Fig. 2:**
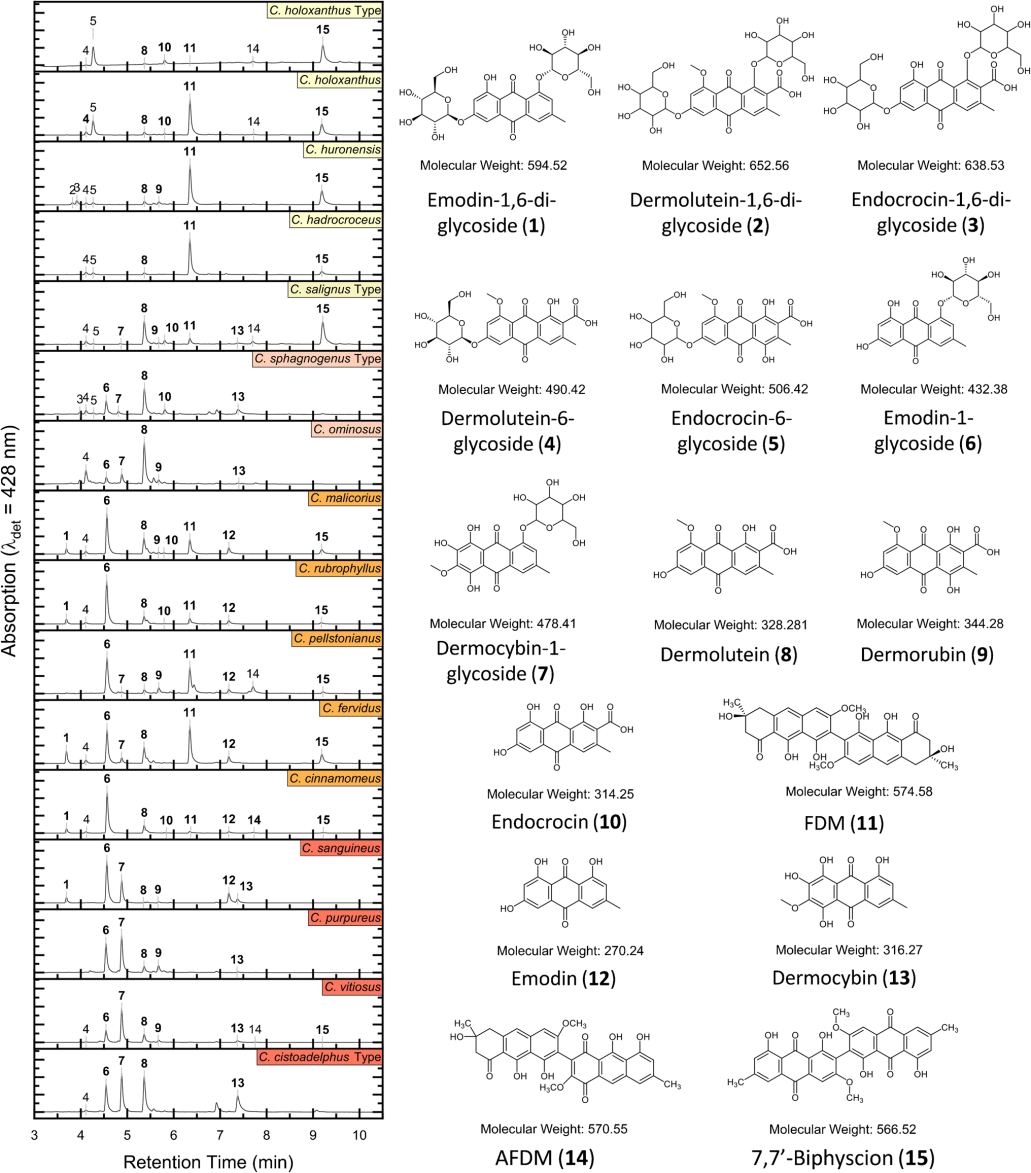
Pigment chromatograms of 16 dermocyboid *Cortinarius* species, measured at 428 nm; the retention time is printed against the height of the peaks; numbers above peaks indicate pigments, names, assigned numbers and chemical structure visible on the right; colouration of box surrounding the taxon name indicate pigment groups

The following compounds were found in different concentrations in the *Cortinarius* species investigated in this study: emodin-1,6-di-glycoside (**1**), dermolutein-1,6-di-glycoside (**2**), endocrocin-1,6-di-glycoside (**3**), dermolutein-6-glycoside (**4**), endocrocin-6-glycoside (**5**), emodin-1-glycoside (**6**), dermocybin-1-glycoside (**7**), dermolutein (**8**), dermorubin (**9**), endocrocin (**10**), flavomannin-6.6’-dimethyl-ether (FDM) (**11**), emodin (**12**), dermocybin (**13**), anhydroflavomannin-9,10-chinon-6,6’-dimethylether (AFDM) (**14**), 7,7’-biphyscion (**15**). For pigment profiles and chemical sturctures of annotated pigments see Fig. 2.

**Pigment groups—**Based on their pigment profiles, the species studied here could be arranged into the following four pigment groups (Fig. 2). Pigment group 1 – This so called Croceus-pigment group was detected in *C*. *hadrocroceus*, *C*. *holoxanthus*, *C*. *huronensis*, and *C. salignus.* The characteristic yellow colour of the lamellae and the whole fruitbody, is due to the high content of FDM (**11**). Pigment group 2 – This Malicorius-pigment group could be assigned to *C. cinnamomeus, C*. *fervidus*, *C*. *malicorius, C*. *pellstonianus, *and* C*. *rubrophyllus.* These are all species with an orange to fire-coloured velum, and orange to reddish lamellae. Species share the presence of pigments emodin-1-glycoside (**6**), dermolutein, FDM (**11**), emodin, and 7,7’-biphyscion (**15**). *C*. *malicorius* and *C*. *rubrophyllus* have a very similar pigment profile, with minute differences in pigment traces. *C. pellstonianus* lacks pigments emodin-1,6-di-glycoside (**1**) and dermolutein-6-glycoside (**4**) but has pigment AFDM (**14**). *C. fervidus* additionally has pigment dermocybin-1-glycoside (**7**), which places it closer to the Sanguineus- and the Ominosus-pigment group. Pigment group 3 – The Ominosus-pigment group was defined for *C*. *ominosus* and *C*. *sphagnogenus.* In this group the lamellae are red, but the main colouration of the pileus and the stipe is brownish. Specimens have neither FDM (**11**) nor its oxidation-products, but they have pigments emodin-1-glycoside (**6**), dermocybin-1-glycoside (**7**), and dermolutein (**8**). Additionally, they do have dermolutein-6-glycoside (**4**) and a few traces of other pigments. Generally, one could say that they seem to have many different pigments, although in lower substance quantity levels. Pigment group 4 – The Sanguineus-pigment group was detected in *C*. *cistoadelphus*, *C*. *purpureus*, *C*. *sanguineus* var. *aurantiovaginatus*, and *C*. *vitiosus.* The red fruit body colour is mainly caused by dermocybin (**13**), but in addition, dermocybin-1-glycoside (**7**) is also characteristic.

### Correlation between phylogeny and pigment type

**Fig. 3:**
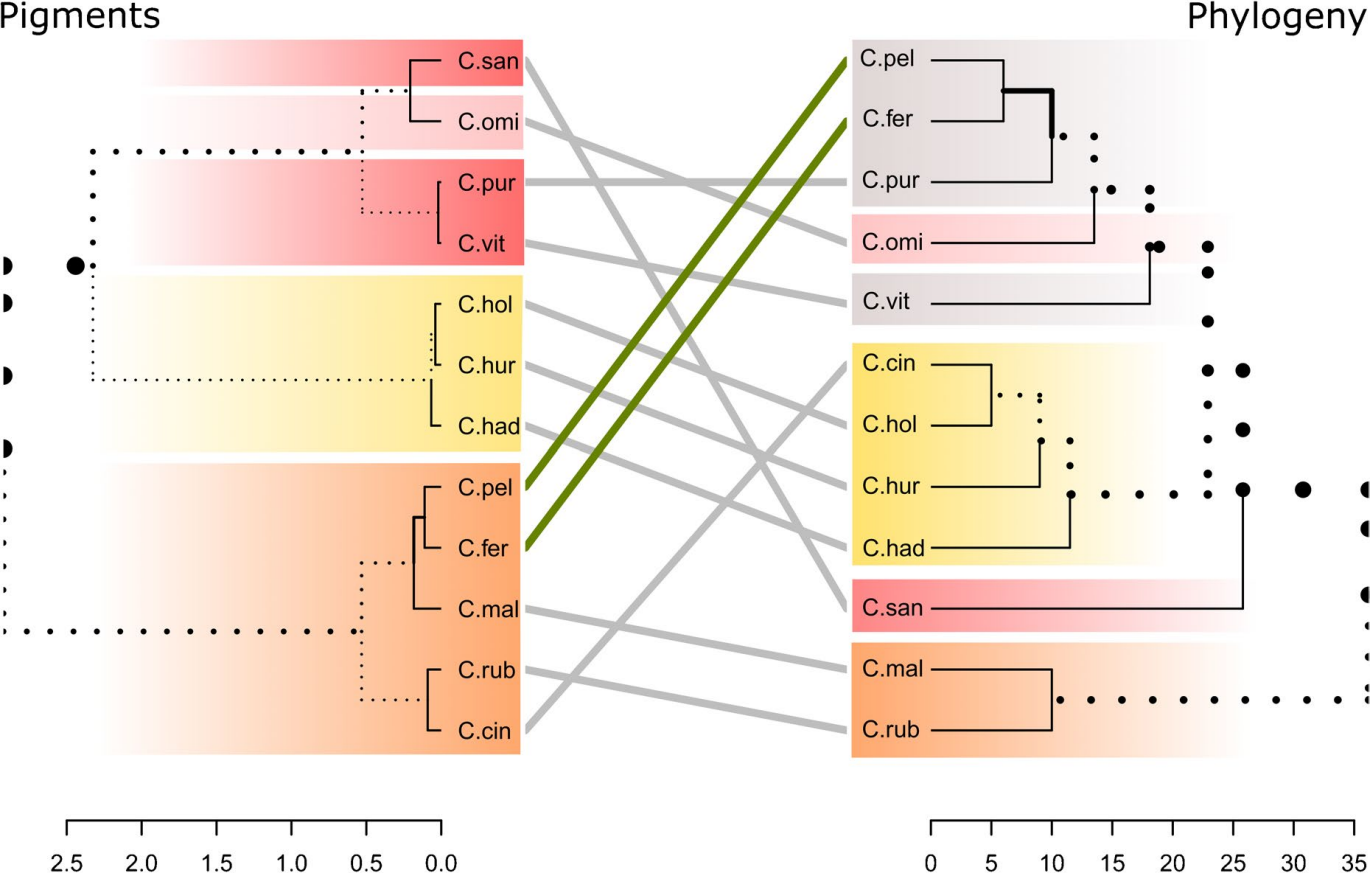
Correlation between pigment profiles (left) and rDNA-based ITS phylogeny (right) of different *Cortinarius* species; green lines indicating a highly significant correlation. Abbreviations: C.cin: *C. cinnamomeus*, C.fer: *C. fervidus*, C.had: *C hadrocroceus*, C.hol: *C. holoxanthus*, C.hur: *C. huronensis*, C.mal: *C. malicorius*, C.omi: *C. ominosus*, C.pel: *C. pellstonianus*, C.pur: *C. purpureus*, C.rub: C. *rubrophyllus*, C.san: *C. sanguineus*, C.vit: *C. vitiosus*. Solid and dotted lines indicate the level of split similarity between both dendrograms

Closely related species mainly have a similar pigment composition as shown in the tanglegram (Fig. 3). Phylogenetic distances (Bray Curtis) are weakly correlated to differences in pigment composition (Correlation coefficient = 0.199, p = 0.019). The placement of single species is not always in accordance between pigment groups and clades. E.g. the phylogenetically closely related *C. malicorius* and *C. rubrophyllus* can be separated by their pigment profiles. *C. cinnamomeus* is phylogenetically in the C. croceus clade, but falls into the Malicorius pigment group, which better reflects the colouration of its fruiting bodies and its spore size.

### Basidiospore morphology

**Fig. 4:**
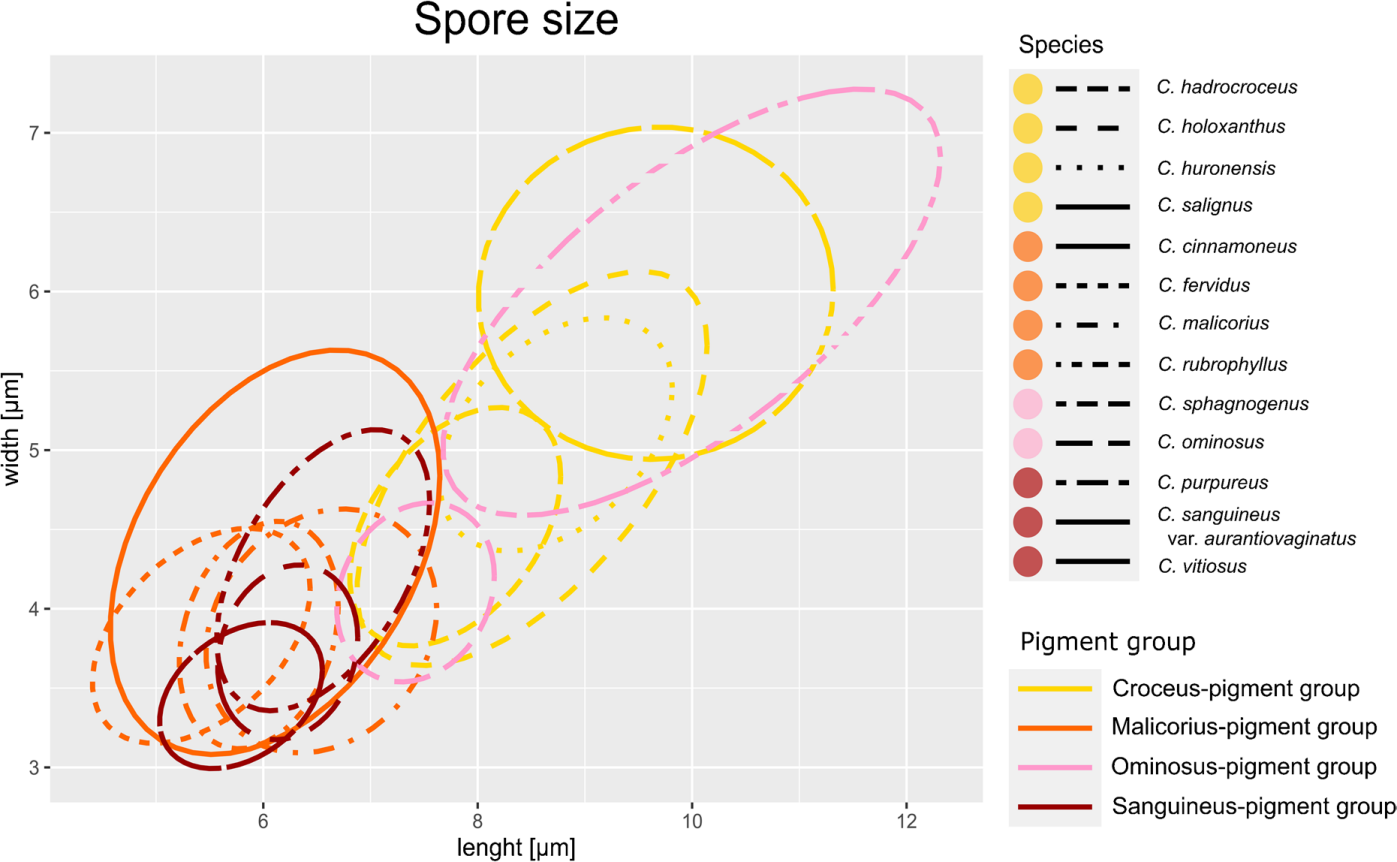
Basidiospore size of *C. cinnamomeus*, *C. fervidus*, *C. hadrocroceus*, *C. holoxanthus*, *C. huronensis*, *C. malicorius*, *C. ominosus*, *C. purpureus*, *C. rubrophyllus*, *C. salignus*, *C. sanguineus*, and *C. vitiosus*; the ellipses are placed based on scatter diagrams and contain 95% of the spore measurements of each species (n ≥ 30); x-axis: length of spores in µm; y-axis: width of spores in µm; the colours of the ellipses indicate the pigment groups; coloured dots beside the species names indicate the pigment-group

Basidiospore size and morphology differed between most of the examined species (Table 1, Fig. 4, additional material Fig. 7), but there were no statistically significant differences in spore size in phylogenetic closely related species or species of the same pigment group. This implies, that the characters spore size and shape alone cannot unambiguously delimitate closely related taxa. Interestingly, species belonging to the same clade or pigment-group often had spores of the same size range and shape:

**Table 1:** Basidiospore sizes measured for dermocyboid *Cortinarius* species

*Species*	lenght [µm]	width [µm]	Q	ornamentation
*C. cinnamomeus*	(4.5) 6.1 ± 0.69 (7.6)	(3.2) 4.3 ± 0.56 (5.4)	1.42 ± 0.19	finely to moderate verrucose
*C. cistoadelphus*	(6.0) 7.5 ± 0.82 (9.2)	(3.7) 4.7 ± 0.52 (6.0)	1.52 ± 0.21	slightly verrucose
*C. croceus*	(7.5) 8.7 ± 0.58 (10.4)	(4.1) 5.2 ± 0.48 (6.7)	1.66 ± 0.18	punctate, verrucose
*C. fervidus*	(4.4) 5.5 ± 0.53 (6.9)	(3.3) 3.9 ± 0.35 (4.9)	1.43 ± 0.14	fine and sparsely dense verrucose
*C. hadrocroceus*	(6.4) 7.8 ± 0.55 (9.2)	(4.1) 4.6 ± 0.33 (5.4)	1.69 ± 0.12	finely verrucose
*C. holoxanthus*	(7.0) 8.0 ± 0.46 (9.0)	(4.0) 4.5 ± 0.27 (5.1)	1.78 ± 0.12	finely to moderately verrucose
*C. holoxanthus* Type	(7.4) 9.1 ± 0.56 (10.5)	(4.3) 5.3 ± 0.44 (6.2)	1.72 ± 0.19	finely to moderately verrucose
*C. huronensis*	(7.9) 8.7 ± 0.48 (9.9)	(4.1) 5.1 ± 0.36 (6.1)	1.72 ± 0.12	finely warty, rugose to verrucose
*C. malicorius*	(5.7) 6.6 ± 0.44 (7.4)	(3.3) 3.9 ± 0.35 (4.8)	1.53 ± 0.13	finely verrucose
*C. ominosus*	(6.8) 7.5 ± 0.34 (8.2)	(3.7) 4.1 ± 0.25 (4.7)	1.82 ± 0.12	finely verrucose
*C. pellstonianus*	(7.1) 8.2 ± 0.51 (9.3)	(4.5) 5.3 ± 0.33 (6.1)	1.55 ± 0.14	finely verrucose to verrucose
*C. purpureus*	(5.7) 6.6 ± 0.42 (7.2)	(3.6) 4.2 ± 0.39 (5.1)	1.56 ± 0.13	smooth to very finely verrucose
*C. rubrophyllus*	(5.2) 6.0 ± 0.39 (7.1)	(3.3) 3.9 ± 0.35 (4.9)	1.70 ± 0.17	finely ornamented
*C. salignus*	(8.0) 9.6 ± 0.62 (10.8)	(5.2) 6.0 ± 0.45 (6.9)	1.61 ± 0.16	very finely verrucose
*C. sanguineus*	(5.6) 6.3 ± 0.33 (7.3)	(3.1) 3.7 ± 0.26 (4.6)	1.68 ± 0.13	moderately verrucose
*C. sphagnogenus*	(8.1) 10.0 ± 0.97 (11.8)	(4.3) 5.9 ± 0.59 (7.0)	1.70 ± 0.15	distinctly warty
*C. vitiosus*	(5.1) 5.8 ± 0.35 (6.7)	(2.9) 3.5 ± 0.21 (3.8)	1.68 ± 0.11	moderately verrucose

The four species with yellow lamellae, phylogenetically belonging to the C. croceus clade, or chemically to C. croceus-pigment group had the largest spores (*Cortinarius salignus*, *C*. *huronensis, C*. *holoxanthus*, and *C*. *hadrocroceus*). Spore values of this group are significantly different from values measured in the Malicorius-pigment group (length p < 2e-16, width p < 2e-16, Q p < 2e-16), and in the Sanguineus-pigment group (length p < 2e-16, width p < 2e-16, Q p = 0.016). The spore values of the Croceus-pigment group are not statistically different from *C. sphagnogenus,* which belongs to the Ominosus-pigment group, which is in accordance with the phylogenetic sistergroup relationship of this species to C. croceus clade. (Fig. 1).

*C*. *malicorius* and *C*. *rubrophyllus* (C. malicorius clade), with close resembling orange brownish fruiting body colours, have small spores. *C*. *malicorius* has significantly longer spores than *C. rubrophyllus* (p = 0.013) and thus a different Q value (p = 0.02188) (Fig. 4). The other species, assigned to the Malicorius-pigment group did not differ significantly in spore size among each other. They all differ in Q values from species of the Sanguineus-pigment group highlighting the fact that the *C*. *vitiosus*, *C*. *sanguineus*, and *C*. *purpureus* have more roundish spores.

## Taxonomy

We aimed at providing an aid on how to delimit the most abundant species occurring mainly in the alpine area from closely related species. We therefore present a dichotomic identification key including 28 European *Dermocybe* spp. based on our data and data from other authors (Moser 1978; Knudsen 2008; Niskanen et al. 2012; Soop 2021). We are aware of the fact that morphology-based identification is very difficult in these taxa: the identification success depends very much on the availability of young basidiomes with well-developed characters.

The analysed *Dermocybe* collections fall into 19 distinct taxa, which we will further address in this study. Interestingly, *C. croceus* was only found once, and *C. semisanguineus* was never found. These two species are obviously often misidentified in these habitats. Instead, we re-discovered several “forgotten” species, which were earlier described from the area or similar habitats, but rarely reported. The assumption of abundance is hereby made only by this study and is therefore mainly reflecting subalpine *Picea abies* forests. We propose one nomenclatural novelty for *C. cistoadelphus*. Furthermore, if no English original description was available, we provide a detailed description—with when available in other languages, also detailed microscopic observations—based on both the original descriptions, complemented with our observations.

### The Cortinarius croceus clade

**Fig. 5:**
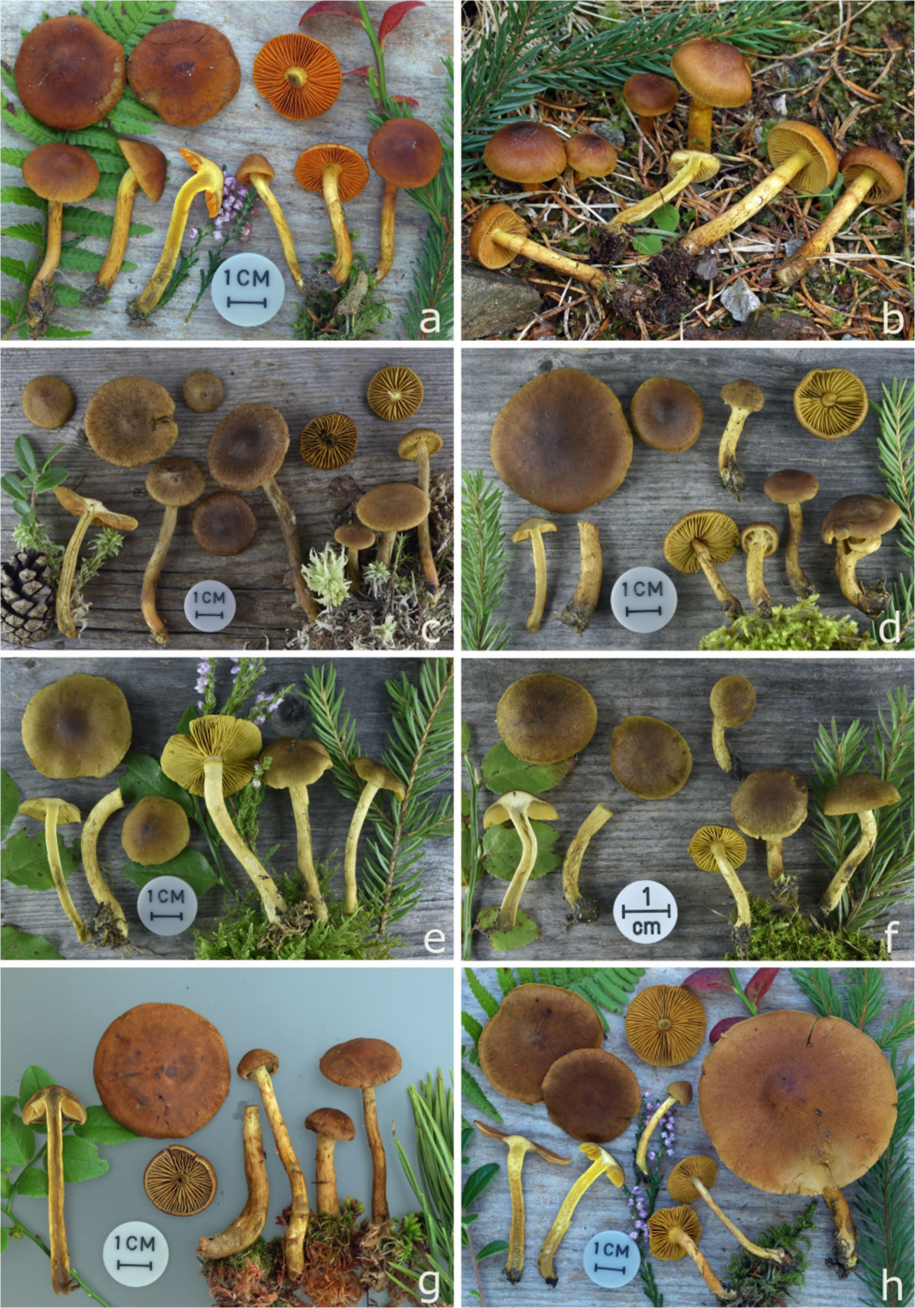
European *Cortinarius* subgenus *Dermocybe*: **a** *C. cinnamomeus* (TG2010058), **b** *C. polaris* (TG2008232), **c** *C. huronensis* (TG2018100), **d** *C. hadrocroceus* (TG2022027), **e** *C. holoxanthus* (2022019), **f** *C. croceus* (TG2022099), **g** *C. davemalochii* (TG2014038), **h** *C. incognitus* (TG2005237)

Specimens of this clade have fruiting bodies with yellow colours (Fig. 5): the pileus is yellow brown to brown, in some species yellow when young, the lamellae are mostly bright yellow, the stipe is more or less yellow and the context is yellow to olive yellow in stipe. Basidiospores of most species in this clade are medium-sized to rather large, but cover a wide range of 4.5–11 µm in length and 3.2–7 µm in width, with Q values from 1.42–1.78 due to the inclusion of the small-spored *C. cinnamomeus*.

***Cortinarius croceus* (Schaeff.) Gray.** A natural arrangement of British plants 1: 630 (1821) [MB#194804] (Fig. 5f).

*Basionym*: *Agaricus croceus* Schaeff. 1774, [MB#476963]; Sanctioning citation: Fr., *Syst. mycol.* **1**: 229 (1821).

*Type*: **Finland**, Ahvenanmaa, Vårdö commune, Sandö, near Sandö sund, *Pinus sylvestris* heath forest on sandy soil; 26 Oct 2006, leg. et det.: K. Liimatainen & T. Niskanen; epitype 06–295 (H6031266); GenBank NR131863.

*Description*: *Pileus* 15–40 mm; at first conical, then low conical to convex to almost plane with a somewhat acute umbo; pileus surface finely felty, brown with brownish yellow margin, when older whole pileus more red. *Lamellae* at first yellow, then orange-brown to cinnamon-brown. *Stipe* 30–55 mm × 3–6 mm (at apex); cylindrical; yellow. *Universal veil* pale somewhat olivaceous brown, rather sparse and forming several incomplete girdles on the stipe. *Basal mycelium Context* in pileus brownish olivaceous yellow, in most of the stipe yellow, base dark brown. *Odour* not available*.* No *UV*-fluorescence. *Macrochemical reaction* not available. *Taste* not available. *Basidiospores* (7.5) 8.7 ± 0.58 (10.4) x (4.1) 5.2 ± 0.48 (6.7); Q = 1.66 ± 0.18; punctate, verrucose.

*Pigments:* Not studied.

*Habitat*: In *Pinus* forests on sandy soil.

*Distribution*: In Europe and North America.

*Specimens examined*: **Italy:** Trentino: Rasun di Sopra, 10. Oct 2022, IBF20220178, Genbank OQ549982.

Note: *C. croceus* is an epithet which is often misapplied for *Cortinarius* species with yellow lamellae. This is notable through many misidentified *C. croceus* sequences on databases. Morphologically, it is very difficult to differentiate the species of the C. croceus clade, as many of them look quite similar (Fig. 5).

***Cortinarius hadrocroceus* Ammirati, Niskanen, Liimat. & Bojantchev.** Index Fungorum 197: 2 (2014). [MB#550834] (Fig. 5d).

*Type*: **Canada**, Quebec, along road 347 between Notre-Dame-de-la-Merci and Saint-Come Forets, Quebec plantation, under *Pinus* on sandy soil; N 461555 W 735537; 22 Sep 2010; leg. et det.: T. Niskanen holotype H: Niskanen 10–122, isotype NY; GenBank KP087978.

*Description*: *Pileus* 20–45 mm; hemispherical to low convex to almost plane with a low umbo; pileus surface fibrillose felty, dark brown at the centre, when young margin pale olivaceous yellow, later brown to red brown. *Lamellae* medium spaced; at first olive yellow, later light olive brown. *Stipe* 30–65 mm × 3–6 mm at apex; cylindrical or slightly enlarged at the base; pale-yellow. *Universal veil* brown, sparse, forming several incomplete girdles on the stipe. *Basal mycelium* yellow. *Context* pale yellow, in fresh basidiomes in the pileus pale olivaceous, in lower part of the stipe olive. *Odour* in lamellae indistinct. *Macrochemical reaction* not available. No *UV*-florescence. *Basidiospores* (6.9) 7.8 ± 0.43 (8.9) µm x (3.9) 4.5 ± 0.32 (5.2) µm; Q = 1.74 ± 0.12 (n = 30); amygdaloid; moderately verrucose.

*Pigments*: dermolutein-6-glycoside (**4**) (traces), endocrocin-6-glycoside (**5**) (traces), dermolutein (**8**) (traces), FDM (**11**), 7,7’-biphyscion (**15**) (traces).

*Habitat*: Associated to *Pinus* spp. or *Picea* on sandy soil, but also to *Arctostaphylos uva-ursi* in subalpine and alpine zones.

*Distribution*: Widespread—known from Europe (Austria, Estonia and Italy) and North America (Canada and USA).

*Specimens examined*: **Austria:** Tirol, Bogner Aste, 13. Oct 2020, leg: L. Huymann, IBF20200027, Genbank MW880249; Ellbögen, 22. Aug 2021, leg: L. Huymann, IBF20210122, GenBank OL712396; Mutters, 10. Aug 2021, leg: L. Huymann, IBF20210114, GenBank OL712393; **Italy:** Trentino: Tesido, 03. Sep 2022, IBF20220175, GenBank OQ549979; Tesselberg, 25. Sep 2009, leg.: G. Turrini, IBF20090145, GenBank OQ549958.

*Notes*: *C*. *hadrocroceus* is frequently misidentified as *C. croceus,* but can be distinguished based on the brown universal veil and the brown pileus colour. Its pigmentation profile fits best to the Croceus-pigment group, but differs clearly by the absence of pigments **9**, **10**, and **14**. *Cortinarius hadrocroceus* differs from other closely related species with yellow lamellae, like *C. croceus*, by the following combination of characters: pileus dark brown at the center, pileus margin when young pale olivaceous yellow, lamellae yellow with at least slightly greenish tinge, young olive yellow, later light olive brown; stipe pale-yellow, with brownish veil girdles. It can be characterized by comparatively robust basidiomata.

***Cortinarius holoxanthus* (M.M. Moser & I. Gruber) Nezdojm. **Examination generis *Cortinarius* Fr. in URSS. VII. Conspectus subgeneris *Dermocybe* (Fr.) Fr. Novosti Sistematiki Nizshikh Rastenii. 17:54 (1980). [MB#118587] (Fig. 5e).

Basionym: *Dermocybe holoxantha* M.M. Moser & I. Gruber, Zeitschrift für Pilzkunde 35: 75 (1969) [MB#329814].

*Type*: **Austria**, Gnadenwald, St. Martin, coniferous forest (*Pinus*, *Picea*); 21 Sep. 1965; leg: Gruber; IBF19650150; GenBank OL712385.

*Description: Pileus* 30–40 mm; initially convex or obtusely umbonate to depressed later; pileus surface appearance fibrillose to somewhat fine fibrous scaly, sub-squamulose towards the pileus margin with raised small scales; not hygrophanous; initially yellow, then yellow with olive-brown tones towards the centre, later yellow with a darker apex as scales showing discolouration with age changing uniformly orange-red. *Lamellae* adnate; narrow, with eroded, finely yellow sawed edge; young uniformly yellow (same colour as pileus), later becoming more brownish, but always with a yellow edge. *Stipe* narrow, mostly cylindrical; base sinuous or bent, attenuated or bulbous enlarged; yellow with base enveloped by whitish, entirely pale lemon to olive-yellow, with basal mycelial tomentum. *Universal veil* yellow, thus often not visible on the stipe, or also conceivable as longitudinally arranged veil remnants, in the lower half of the stipe as reddish-brown appressed fibrillae. *Context* pale olive-coloured in the pileus, olive-yellow to darker towards the central part of the stipe, base also dark olivaceous. *Odour* herbaceous to radish. *Taste* mild. *Macrochemical reaction* with KOH blackish-red on pileipellis; wine-red on veil, outer covering of stipe and flesh; immediate blood-red on lamellae. No *UV*-florescence. *Basidiospores* (7.0) 8.0 ± 0.46 (9.0) µm x (4) 4.5 ± 0.27 (5.1) µm; Q = 1.78 ± 0.12 (n = 30); elliptic to subamygdaloid; finely to moderately warty. *Basidiospores* from the holotype (7.4) 9.1 ± 0.56 (10.5) µm x (4.3) 5.3 ± 0.44 (6.2) µm; Q = 1.72 ± 0.19 (n = 32). *Basidia* 17–35 × 7–8 µm, (bi)-tetra-sporic, cylindrical-clavate, sinuate; yellowish-brown intracellular pigment reddening in 5% KOH detected. *Marginal cells* present and abundant; simple to articulated with two or three overlapping elements and with cylindrical-clavate terminal element (10–40 µm × 5–8 µm). *Clamp connections* present in all tissues. *Cuticle suprapellis* composed of cylindraceous hyphae (× 3–10 µm); with brownish encrusting parietal pigment; raised elements in outer marginal area of pileus, free cylindraceous or attenuated terminals. *Subcutis* consisting of vesiculate-swollen elements (× 25 µm); endowed with brownish encrusting parietal pigment. *Hypodermis* with intracellular pigment in the form of granules or distributed in amorphous masses; reddening with 5% KOH.

*Pigments:* dermolutein-6-glycoside (**4**) (traces), endocrocin-6-glycoside (**5**), dermolutein (**8**) (traces), dermorubin (**9**), endocrocin (**10**) (traces), FDM (**11**), AFDM (**14**) (traces), 7,7’-biphyscion (**15**).

*Habitat*: Growing in mossy damp environments, often associated with young *Picea*.

*Distribution*: In Europe (known from Austria and Italy).

*Specimens examined*: **Austria:** Tirol: Bogner Aste; 13. Oct 2020, leg.: L. Huymann, IBF20200029, GenBank MW880251; Gnadenwald bei St. Martin; 21. Sep 1965, leg.: I. Gruber, IBF19650150, GenBank OL712385; Lans, 03. Sep 2019, leg.: L. Huymann, IBF20190009-2; 03. Sep 2019, leg.: L. Huymann, IBF20190009-2; 30. Sep 2019, leg.: L. Huymann, IBF20190009-3; 03. Sep 2021, leg.: L. Huymann, IBF20210165, GenBank OL712407; 08. Sep 2020, leg.: L. Huymann, IBF20200030, GenBank MW880252; 01. Sep 2021, leg.: L. Huymann, IBF20210144; 03. Sep 2021, leg.: L. Huymann, IBF20210166; Mutters, 08. Sep 2019, leg.: L. Huymann, IBF20190009-4; 04. Oct 2020, leg.: L. Huymann, IBF20200028, GenBank MW880250; 14. Sep 2020, leg.: L. Huymann, IBF20200031, GenBank MW880253; 28. Sep 2020, leg.: L. Huymann, IBF20200070, GenBank OL712405; 30. Aug 2021, leg.: L. Huymann, IBF20210172; **Italy:** Trentino: Chienes, 08. Sep 2020, leg.: G. Turrini, IBF20200080, GenBank OQ549976; Falzes, 20. Aug 2022, leg.: G. Turrini, IBF20220174, GenBank OQ549978; Falzes-Unterberg, 27. Oct 2013, leg.: G. Turrini, IBF20130236, GenBank OQ549967.

*Notes*: For a long time, it was unclear if *C. holoxanthus* as nearly no findings were reported (Fellin et al. 2020) apart from its lack in modern identification keys. Morphologically, *C*. *holoxanthus* is a typical representative of the C. croceus clade (Fig. 5), where it resembles *C. croceus, C. hadrocroceus,* and *C. salignus.* Additional to the ITS data, the pigment pattern had 8 characteristic differences in thin layer chromatogram, thus providing further evidence for *C*. *holoxanthus* being a distinct species (Gruber and Moser 1969).

***Cortinarius huronensis* (Ammirati & A.H. Sm) Ammirati & A.H. Sm.** Michigan Bot. 11(1): 20 (1972) [MB#419314] (Fig. 5c).

*Basionym*: *Cortinarius huronensis* Ammirati & A.H. Sm., The Michigan Botanist 11: 20 (1972), *Synonyms*: *Dermocybe huronensis* (Ammirati & A.H. Sm.) Ammirati, Mycotaxon 33: 439 (1988); *Cortinarius palustris* var. *huronensis* (Ammirati & A.H. Sm.) Høil., Opera Botanica 71: 90 (1984), *Dermocybe palustris* var. *huronensis* (Ammirati & A.H. Sm.) Tartarat, *Fl. Analyt. Cortin.* (Dauphiné-Savoie): 26 (1988).

*Holotype*: **USA**, Michigan, Marquette Co., Beaver Lake, scattered in sphagnum under conifers; 23 Sep 1970; leg. et. det.: J. F. Ammirati; holotype MBT158902, MICH:Ammirati 5403; GenBank PP001387.

*Description: Pileus* 13–45 mm; convex, plane or umbonate; pileus surface with radially fibrillose felty, sometimes with minute, appressed scales; dark yellow brown to hazel. *Lamellae* yellow when young, more ochre when older. *Stipe* 32–100 mm × 2–6 mm; light-yellow to whitish, later darker, with orange-reddish base especially when young or bruised. *Universal veil* grey-brown, remnants covering the stipe. *Context* pale yellow in the pileus, becoming watery olive, olivaceous yellow in the stipe, in the base with reddish hue. *Macrochemical reaction* with KOH on pileus reddish to red brown; on lamellae red brown to carmine. *UV*-florencence of lamellae under 350 nm light yellowish-orange. *Basidiospores* (7.9) 8.7 ± 0.48 (9.9) µm x (4.1) 5.1 ± 0.36 (6.1) µm; Q = 1.72 ± 0.12 (n = 30); ovoid to elliptical; finely warty. *Basidia* can contain a yellow granular pigment.

*Pigments*: dermolutein-1,6-di-glycoside (**2**) (traces), endocrosin-1,6-di-glycoside (**3**), dermolutein-6-glycoside (**4**) (traces), endocrocin-6-glycoside (**5**) (traces), dermolutein (**8**) (traces), dermorubin (**9**) (traces), FDM (**11**), 7,7’-biphyscion (**15**).

*Habitat*: mostly among *Sphagnum* in fens, bogs, or swampy forests, moist humus, together with *Picea*, *Pinus*, *Betula*, and rarely *Salix.* Common in hemi boreal arctic and alpine, and rarer in temperate climate.

*Distribution*: In Europe (known from Austria, Denmark, Finland, Island, Italy, Norway, Romania, and Sweden).

*Specimens examined*: **Austria:** Tirol: Bogner Aste, 13. Oct 2020, leg.: L. Huymann, IBF20200023, GenBank MW880255; 13. Oct 2020, leg.: L. Huymann, IBF20200024, GenBank MW880256; Lans, 01. Sep 2021, leg.: L. Huymann, IBF20210153; 21. Sep 2020, leg.: L. Huymann, IBF20200026, GenBank MW880258; Mutters, 02. Oct 2020, leg.: L. Huymann, IBF20200022, GenBank MW880254; 04. Oct 2020, leg.: L. Huymann, IBF20200025, GenBank MW880257; 04. Oct 2020, leg.: L. Huymann, IBF20200025; Patsch, 20. Sep 2018, leg.: L. Huymann, IBF20180121, GenBank MW880259; **Italy**: Trentino: Corti, 22. Aug 1999, leg.: G. Turrini, IBF19991067, GenBank OQ549947; St. Lorenzo di Sebato (St. Lorenzen), 18. Sep 2015, leg.: G. Turrini, IBF20150233, GenBank OQ549971; **Romania**: Transilvania: Verasvíz, 17. Oct 2018, leg.: G. Turrini, IBF20180237, GenBank OQ549975.

*Notes*: *Cortinarius huronensis* is the correct name for the above-mentioned collections. *C*. *huronensis* was often considered as a synonym for *C*. *chrysolitus* Kaufman, or as a variety of *C. chrysolitus* (Kuhnert-Finkernagel and Peintner 2003). However, based on our phylogenetic study including type specimen, *C*. *chrysolitus* is a distinct species. The ITS-sequence of the *C*. *huronensis* type falls into a well-supported clade (0.99 BPP) with *C*. *aurantiobasis,* whilst the type of *C*. *chrysolitus* (JX045672) is closer to *C*. *vitiosus* or *C*. *cruentiphyllus*. We noted 15 differences between the ITS sequence of the types of *C*. *chrysolitus* and *C*. *huronensis*. The other sequences of *C*. *huronensis,* created in this study, had up to 16 differences to *C*. *chrysolitus*. Morphologically, *C. huronensis* differs from *C*. *chrysolitus. C*. *chrysolitus* has a dark brown pileus, light greenish lamellae, and the alkaline reaction is red brown to brown on the lamellae, furthermore it grows under *Pinus* (Soop 2021). *C*. *huronensis* has paler, more dark yellowish brown to olive hazel brown pilei, more yellow to later ochre lamellae, and the KOH reaction is red brown to carmine (Knudsen and Vesterholt 2008). Additionally, *C*. *huronensis* has the characteristically slightly orange-olive to orange red colours at the base of the stipe, which distinguish it from other species in the C. croceus clade (Fig. 5).

***Cortinarius salignus* (M.M. Moser & Gerw. Keller) G. Garnier.** Bibliographie des Cortinaires. P–Z.: 59 (1992) [MB#622261].

*Basionym*: *Dermocybe saligna* M.M. Moser & Gerw. Keller, Zeitschrift für Pilzkunde 43: 207 (1977).

*Type*: **Sweden**, Småland, Femsjö, on acidic soil in swampy places under *Salix aurita, S. cinerea;* 19 Sep 1998; leg. et det.: M. Moser; holotype IBF19760208; GenBank OL712388.

*Description*: *Pileus* 1–4.5 cm; blunt conical; yellow-olive, later more olive, old specimens also with interspersed slightly red-brownish to umbra-brownish tones and somewhat marbled, wet with umbra-brown spots; young with with radial fibres, older with very fine but appressed scales, specially towards the margin. *Lamellae* yellow; edges notched; broadly adnate; slightly narrow. *Stipe* 30–100 mm × 1–4 (–5) mm, base 7 mm broad; more or less cylindric to bent, somewhat thickened at the base; lively yellow (Expo 88A), base covered with light olivaceous mycelium (Rigway XVII Pele Chalcedony Yellow to Light Chalcedony Yellow), in old specimens the stipe colouration gets mixed with a reddish-brown tinge. *Context* in the pileus watery olive yellow, in the stipe lively yellow. *Odour* insignificant. *Taste* mild. *Macrochemical reaction* with KOH lamellae orange brown; pileipellis dark red brown. No *UV*-fluorescence, only basal mycelium slightly yellow under 350 nm. *Basidiospores* (8) 9.6 ± 0.62 (10.8) µm x (5.2) 6.0 ± 0.45 (6.9) µm; Q = 1.61 ± 0.16 (n = 30); elliptical to subamygdaloid; very finely warty. *Basidia* 28–32 µm × 8–9 µm; tetra-sporic. *Sterigma* approx. 5 µm*. Lamella edge* without cheilocystidia, only basidioles and basida. *Cuticle suprapellis* composed of hyphae (5–9 µm), thicker ones shorter (25–35 µm), thinner ones longer (50–60 µm), close to the surface, with c*lamp connections*. *Trama* hyphae with yellow to yellow-brownish intracellular pigments (FDM (**11**)).

*Pigments*: dermolutein-6-glycoside (**4**) (traces), endocrocin-6-glycoside (**5**) (traces), dermocybin-1-glycoside (**7**) (traces), dermolutein (**8**), dermorubin (**9**) (traces), FDM (**11**), dermocybin (**13**) (traces), 7,7’-biphyscion (**15**).

*Habitat*: Damp or swampy places with *Salix* (*S. aurita*, *S. cinerea*) on strongly acidic soil.

*Distribution:* In Europe (known from Italy, Finland, Norway, Sweden, and the United Kingdom).

*Specimens examined*: **Italy:** Trentino: Stelvio, 15. Aug 2017, leg.: G. Turrini, IBF20170594, GenBank OQ549973; 17. Aug 2019, leg.: G. Turrini, IBF20180236, GenBank OQ549974; **Sweden**: Femsjö: Yaberg, 19. Sep 1998, leg.: M. Moser, IBF19760208, GenBank OL712388.

*Notes*: The holotype of *C. salignus* was newly sequenced for this study. Based on the phylogenetic analysis of ITS sequences, *C*. *salignus* is part of the C. croceus clade, where it falls in a lineage together with *C. cinnamomeoluteus* (MN751102, U56040) (with 0.77 PP) and *C. ferruginosus*. Moser (1976) considered *C. salignus* on species level because of the characteristic pigment pattern and habitat. Morphologically it is closely related to *C. holoxanthus*, from which it mainly differs by the habitat and associated *Salix*. In alpine areas, there is a habitat overlap with *C. polaris,* leading to misidentification*.* The sequence labelled *C. polaris* (KC842411) (with 1 PP), is such a misidentification and rather represents *C*. *salignus,* as it differs by 11 bases from the ITS-sequence of the *C*. *polaris* type, which also belongs to a different clade.

***Cortinarius sphagnogenus* (M.M. Moser) Nezdojm**. Ad floram Agaricalium partis borealis regionis Krassnojarsk. Novosti Sistematiki Nizshikh Rastenii. 13:111, (1976). [MB#312142].

*Basionym*: *Dermocybe sphagnogena* M.M. Moser, Schweizerische Zeitschrift für Pilzkunde 51: 132 (1973) [MB#283431].

*Type*: **Denmark**, Fünen, Diernaes, Gerup Skov, in *Sphagnum* sp., besides a pond; 22. Sep 1970; leg. et det.: M. Moser; holotype: IBF19700271; GenBank OL712387.

*Description*: *Pileus* 1.5–5.0 cm; young hemispherical, later bluntly cup-shaped to convex, often humped, globous; pileus surface with finely pressed radial fibrous, old sometimes cracked-scaly; ochre to yellow–brown, reddish brown to umber brown, at the edge somewhat buckthorn-brown, middle to cinnamon brown, older specimens pronounced reddish brown. *Lamellae* L = 40–45 mm, l = 3, 4–6 mm wide; 4–6 × pileus flesh thickness, and massively crowded; colour young yellow with an olive tinge, Aniline-yellow, to Primuline-yellow, old with olive tinge and yellow-ochre. *Stipe* 5–10 cm × 3–6 mm; cylindrical, or gradually slightly thickened towards base; young yellow, old with olive tone, brownish towards base, without velum traces. *Universal veil* inconspicuous. *Context* moist watery olive-brownish in pileus, watery olive-yellow in stipe, paling to yellow in cortex. *Odour* absent or very faintly reddish-like. *Taste* mild, slightly and indistinctly reddish-like. *Macrochemical reaction* with KOH not red, at most red-brownish. *UV*-florencence positive bright yellowish orange in in stipe; lamellae and pileus lighter under 350 nm, only little flourenscence under 250 nm (adapted from Moser 1973). *Basidiospores* (8.1) 10.0 ± 0.97 (11.8) µm x (4.3) 5.9 ± 0.59 (6.8) µm; Q = 1.70 ± 0.15 (n = 30); ellipsoidal; distinctly warty. *Basidia* vesicular, 28–30 µm × 7–8 µm; at the sheath clubbed cells, some also bulbous of 20–25 µm × 6–9 µm. *Intercellular pigment* masses yellow–brown; relatively sparse.

*Pigments*: endocrocin-1,6-di-glycoside (**3**) (traces), dermolutein-6-glycoside (**4**), endocrocin-6-glycoside (**5**) (traces), emodin-1-glycoside (**6**), dermocybin-1-glycoside (**7**), dermolutein (**8**), endocrocin (**10**), dermocybin (**13**).

*Habitat:* often between *Sphagnum* bogs, and on lakeshores and other damp mossy forests.

*Distribution:* Austria and Denmark.

*Specimens examined*: **Austria:** Salzburg: Breitenberg, 02. Oct 1999, leg.: G. Turrini IBF19991068, GenBank OQ549948; **Denmark:** Fünen: Diernaes, Gerup Skov, 22. Sep 1970, leg.: M. Moser, IBF19700271, GenBank OL712387.

*Notes*: We analysed the holotype of *C. sphagnogenus,* as there was was a lot of confusion around this species. *C. sphagnogenus* was 1976 recombined by Nezdojmingo and is a valid species, not to be confused with *C. sphagneti* described by Singer 1949 or the 1958 illegitimate described *C. sphagneti* by Orton. *Cortinarius sphagneti* was later addressed as Cortinarius *palustris* f. *sphagneti* (M.M. Moser) Nespiak, *Flora Polska, Grzyby* (1975) (Basidionym: *Dermocybe palustris* var. *sphagneti* M.M. Moser), its current name is *Cortinarius tubarius* Ammirati & A.H. Sm.. These two taxa are sistergroups (Fig. 1).

When first describing *C*. *sphagnogenus*, (Moser 1973) wrote that it is not easy to distinguish it from *C. tubarius*. In our phylogenetic analysis *C*. *sphagnogenus* is a sister to *C*. *tubarius* (Type: PP001390) but shows differences in three bases in the ITS sequence. Morphologically *C*. *sphagnogenus* differs mainly by its less pronounced olive colouration: *C*. *tubarius* has a more olive pileus (olive-yellow brownish, rusty brown olive brown to dark brown) compared to *C*. *sphagnogenus*, whose pileus was described as yellow–brown, rusty-yellow brownish to dark brown and fibrillose. In general, basidiome size they are not significantly different, but *C*. *sphagnogenus* can have a slightly larger pileus while *C*. *tubarius* can have a slightly longer stipe. The stipe of *C*. *tubarius* is olive-greenish, later olive-brown while *C*. *sphagnogenus’* stipe is first yellow and later rusty-brown to olivaceous. *C*. *tubarius* has yellow brownish only slightly warty basidiospores while *C*. *sphagnogenus* basidiospores are rusty-brown and clearly warty. We did not detect C. *tubarius *in our alpine coniferous habitats, although is is reported to occur in similar habitats, namely among *Sphagnum* sp. under *Picea abies* (Moser 1973; Gyosheva and Ganeva 2004).

***Cortinarius cinnamomeus* (L.) Gray.** A natural arrangement of British plants 1: 630 (1821). [MB#197182] (Fig. 5a).

*Basionym*: *Agaricus cinnamomeus* L., Species Plantarum: 1173 (1753).

*Synonyms: Dermocybe cinnamomea* (L.) Wünsche, Die Pilze. Eine Anleitung zur Kenntniss derselben. 125 (1877);

*Flammula cinnamomea* (L.) P. Kumm., Der Führer in die Pilzkunde: 81 (1871).

*Type*: **Sweden**, Ångermanland, Säbrå sn, Innerbrån, young mixed forest; from soil; 11 Sep 1987; leg. et. det.: Tor Erik Brandrud, Håkan Lindström, Hans Marklund & Siw Muskos; SF44851 neotype, CFP623, GenBank NR131816.

*Description: Pileus* 12–60 mm; umbonate to convex; cinnamon-brown, yellow–brown to orange-brown when young, later dark red brown or chestnut brown. *Lamellae* bright orange when young, cinnamon-orange when older. *Stipe* 18–65 mm × 4–10 mm; surface pale yellow, olivaceous yellow or yellow brown base usually orange-red felty. *Universal veil* covering the stipe red brown, ochraceous brown or grey brown. *Context* yellow in the pileus and stipe, lemon yellow at the margin of the stipe, in the centre more ochraceous, becoming olivaceous towards the base of the stipe. *Odour* indistinct. *Macrochemical reaction* not available. No *UV*-fluorescence. *Basidiospores* (4.5) 6.1 ± 0.69 (7.6) µm x (3.2) 4.3 ± 0.56 (5.4) µm; Q = 1.42 ± 0.19 (n = 36); ovoid to amygdaloid; finely to moderate verrucose.

*Pigments*: emodin-1,6-di-glycoside (**1**) (traces), dermolutein-6-glycoside (**4**) (traces), emodin-1-glycoside (**6**), dermolutein (**8**) (traces), endocrocin (**10**), FDM (**11**), emodin (**12**), AFDM (**14**), 7,7’-biphyscion (**15**).

*Ecology*: Associated with *Picea*, *Pinus, Betula*, rarely with other trees, on sandy soil, humus or among mosses, often along roadsides. Summer to autumn.

*Distribution*: In Europe (known from Austria, Denmark, Finland, Italy, Norway and Sweden).

*Specimens examined*: **Austria**: Tirol: Patsch, 20. Sep 2018, leg.: U. Peintner, IBF20180120a; Lans, 21. Sep 2020, leg.: L. Huymann, IBF20200064; IBF20020614; **Italy:** Pistioa: Abetone, 18. Oct 2018, leg.: L. Huymann & U. Peintner, IBF20180177, Genbank MW880248; Trentino: Abruzzo, 28. Sep 2019, leg. G. Turrini, IBF20190153, OM638753; Bagni di Pervalle, 17. Jul 1999, leg.: G. Turrini, IBF19991066, Genbank OQ549946; St. Lorenzo di Sebato, 10. Sep 2005, leg.: G. Turrini, IBF20050704, Genbank OQ549953; 09. Sep 2010, leg.: G. Turrini, IBF20100141, Genbank OQ549959.

*Notes*: The name *Cortinarius cinnamomeus* is wrongly applied as a collective name for dermocyboid Cortinarii with cinnamom-yellow to orange-brownish lamellae. The true distribution of this species remains unknown, and records have to be considered with care. Phylogenetic analysis showed that *C.* *cinnamomeus* falls into the C. croceus lineage, where it has a sister-group relationship to *C*. *uliginosus* (with two sequences: NOBAS5888, NOBAS427). This is in accordance with earlier chemotaxonomical studies, where *C*. *cinnamomeus* was placed into the yellow pigment type (Keller et al. 1988). Our analyses reveal that the *C. cinnamomeus* pigment pattern differs from that of the "yellow" group due to the presence of pigments **1**, **6**, and **12**, as well as low levels of **11** and **15**, and the absence of pigment **5**. Therefore, *C. cinnamomeus* is better classified within the Malicorius-pigmentation group. This species shares morphological characters with both groups: based on the colouration of the lamellae, *C*. *cinnamomeus* (Fig. 5) fits well to Malicorius pigment group, whereas based on the yellow colour of the stipe it fits well with species from the C. croceus clade.

### The Cortinarius malicorius clade

The species of this lineage have orange brownish fruiting bodies with intensely orange or red-brown lamellae, the universal veil has the same intensely orange or red-brown colour, the stipes can be from yellow to orange brown and basidiospores are generally small, with a range of 5.2–7.4 µm in length and 3.3–4.9 µm in width, and Q values from 1.56 to 1.70.

***Cortinarius malicorius* Fr.** Epicrisis Systematics Mycologici: 289 (1838) [MB#219743].

*Synonym*: *Dermocybe malichoria* (Fr.) Ricken, Die Blätterpilze 1:160 (1915) [MB#517896]; *Dermocybe malicoria* (Fr.) Ricken: 160 (1915) [MB#586473].

*Type*: **Sweden**, Småland, Femsjö, the W part of "Flahult skog", close to the cross-road to Boldshult, on needle-mould under old spruces in coniferous wood; 30 Aug. 1943; leg.: Seth Lundell; neotype F-695242 in herb. UPS.

*Description*: *Pileus* 17–50 mm; conical to plane; red brown, yellow–brown or hazel-brown, margin covered with orange veil remnants. *Lamellae* bright orange to ochraceous orange. *Stipe* 24–44 mm × 5–10 mm; brownish. *Universal veil* bright orange; covering the stipe as yellow to bright orange remnants. *Context* olivaceous. *Taste* and *Odour* inconspicuous. *Macrochemical reactions* with KOH context brown red; lamellae bright red. No *UV*-fluorescence. *Basidiospores* (5.7) 6.6 ± 0.44 (7.4) µm x (3.3) 3.9 ± 0.35 (4.8) µm; Q = 1.53 ± 0.13 (n = 30); ovoid to amygdaloid; finely ornamented.

*Pigments*: emodin-1,6-di-glycoside (**1**), dermolutein-6-glycoside (**4**) (traces), emodin-1-glycoside (**6**), dermolutein (**8**), dermorubin (**9**) (traces), endocrocin (**10**) (traces), FDM (**11**), emodin (**12**), 7,7’-biphyscion (**15**).

*Habitat: C. malicorius* grows in coniferous forests, usually with *Picea* and often also with *Alnus*, seldom with other trees, often on rich soil.

*Distribution*: In Europe (known from Austria, Denmark, Finland, France, Italy, Norway, and Sweden).

*Specimens examined*: **Austria:** Tirol: Ellbögen, 24. Aug 2019, leg.: U. Peintner, IBF20190056C, GenBank OL712401; 22. Aug 2021, leg.: U. Peintner, IBF20210124, GenBank OL712398; Grinzens, 25. Aug 2021, leg.: U. Peintner, IBF20210173; Gries am Brenner, 01. Sep 2021, leg.: U. Peintner, IBF20210143; Mutters, 14. Sep 2020, leg.: L. Huymann, IBF20200051, GenBank MW880263; 08. Oct 2020, leg.: L. Huymann, IBF20200071, GenBank OL712404; 10. Aug 2021, leg.: L. Huymann, IBF20210113, GenBank OL712392; 28. Aug 2021, leg.: L. Huymann, IBF20210171; Natters, 22. Aug 2019, leg.: L. Huymann, IBF20190003-2, IBF20190003-1; 08. Oct 2020, leg.: L. Huymann, IBF20200049, GenBank MW880261; 26. Sep 2020, leg.: L. Huymann, IBF20200048, GenBank MW880260; 14. Sep 2019, leg.: L. Huymann, IBF20190009-5; 08. Sep 2020, leg.: L. Huymann, IBF20200050, GenBank MW880262; 21. Sep 2020, leg.: L. Huymann, IBF20200054, IBF20200058; **Italy**: Trentino: St. Lorenzen, 25. Aug 2006, leg.: G. Turrini, IBF20060552, GenBank OQ549955.

*Notes*: *C*. *malicorius* is very similar to* C*. *rubrophyllus*. They are sister-groups and are assigned to the same pigment type. These two species form a well-supported clade (PP/BS = 1/83), and the delimitation to other lineages is highly supported (PP/BS = 1/96). They can be distinguished based on different, characteristic pigment concentrations, which reflect the slightly different colouration of the fruiting bodies (see notes under *C. rubrophyllus*).

**Fig. 6:**
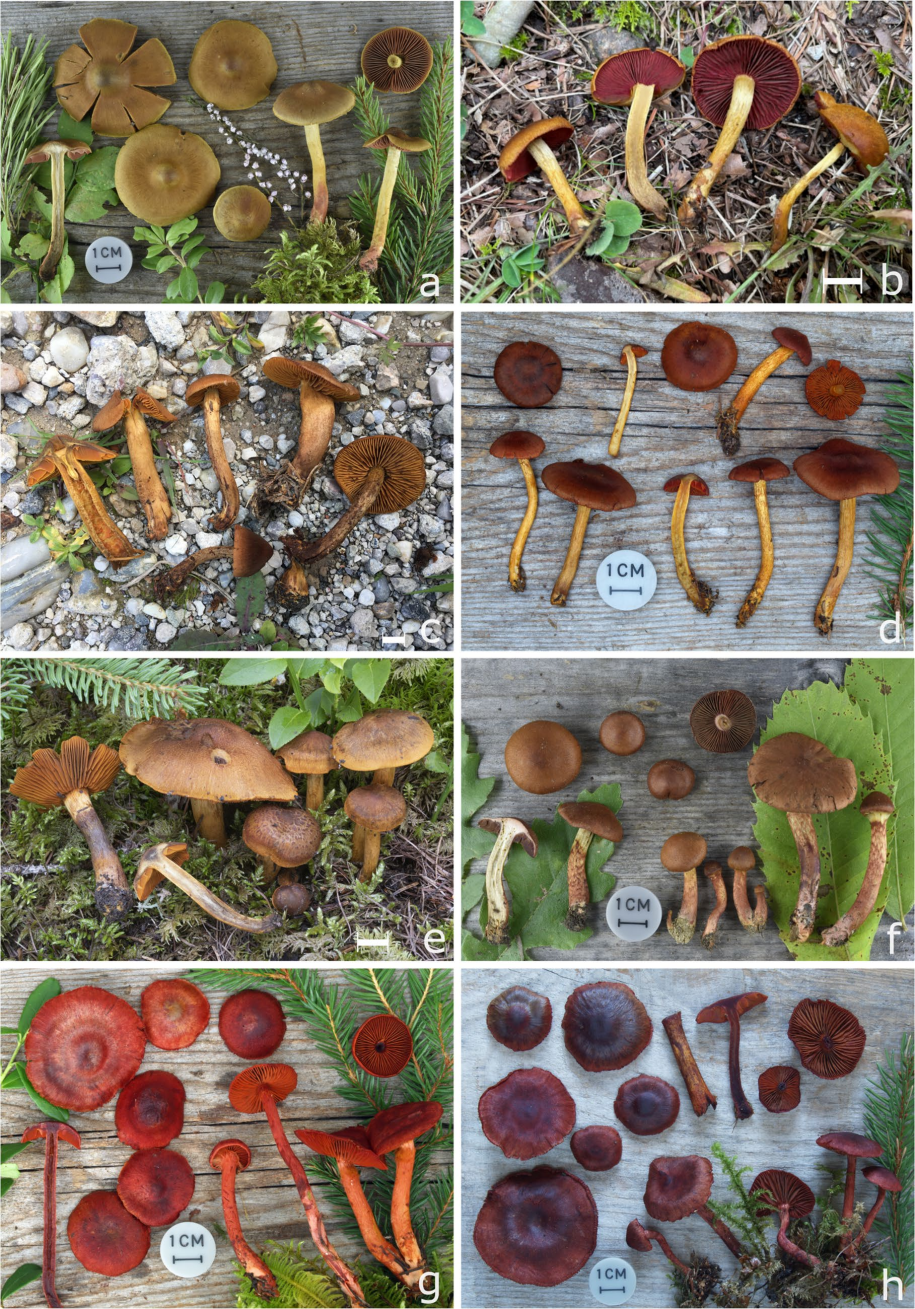
European *Cortinarius* subgenus *Dermocybe*: **a **& **b** *C. ominosus* (TG2011156, IBF20200018), **c** *C. pellstonianus* (IBF20210121), **d** *C. fervidus* (TG2010131), **e** *C. rubrophyllus* (TG2021039), **f** *C. purpureus* (TG2022152), **g** & **h** *C. sanguineus* var. *aurantiovaginatus* (TG2010095, TG2006069); schale bars = 1 cm

***Cortinarius rubrophyllus* (Moënne-Loccoz) Liimat., Niskanen, Ammirati & Dima.** Index Fungorum 196: 3 (2014). [MB#550804] (Fig. 6e).

*Basionym*: *Cortinarius malicorius* var. *rubrophyllus* Moënne-Locc., Atlas des Cortinaires 6: 190 (Fiche 229, Pl. 125) (1994). [MB#446964].

*Type*: **France**, Gallia, region of Poisy (Haute-Savoie), coming from melee, limestone woods, Montagne d'Age; alt.: 600 m; 09 Jul. 1985; leg.: R. Baubet.; holotype no. 234 in herb. GK, GenBank KP087975.

*Description*: *Pileus* brown, chestnut-brown to orange-brown, margin can be brighter through orange veil, older specimens are darker in the centre. *Lamellae* adnate to emarginate; purple red, but of a more sustained red to blood-red; edges do have the same colour and are more or less eroded. *Stipe* bright orange at the apex, carmine at the base. *Universal veil* bright orange covering the pileus and especially abundantly the stipe-base. C*ontext* greenish in the pileus, dirty discoloured at the base of the stipe, lemon yellow at the stipe apex. *Odour* raphanoid. *Macrochemical reactions* with KOH context brown red; lamellae bright red. No *UV*-fluorescence, adapted from (Bidaud et al. 1994) *Basidiospores* (5.2) 6.0 ± 0.39 (7.0) µm x (3.3) 3.9 ± 0.35 (4.9) µm; Q = 1.70 ± 0.17 (n = 30); elliptical; finely ornamented. *Basidia* are short (25–30 µm × 5–7 µm). *Sterile cells* on the edge of the lamellae (diam. 3–6 µm). *Pileipellis* with suprapellis formed by short and wide hyphae measure 5–15 µm, assembled in straightened bundles; with a yellow–brown pigment.

*Pigments*: emodin-1,6-di-glycoside (**1**), dermolutein-6-glycoside (**4**) (traces), emodin-1-glycoside (**6**), dermolutein (**8**) (traces), endocrocin (**10**) (traces), FDM (**11**), emodin (**12**) (traces), dermocybin (**13**), 7,7’-biphyscion (**15**).

*Habitat:* Among young *Picea abies*.

*Distribution:* In Europe (Austria, France, and Italy).

*Specimens examined:* **Austria**: Tirol: Bogner Aste, 14. Sep 2020, leg.: L. Huymann, IBF20200056; 01. Oct 2020, leg.: L. Huymann, IBF20200033, GenBank MW880275; Ellbögen, 24. Aug 2019, leg.: U. Peintner, IBF20190056A, GenBank OL712399, IBF20190056B, GenBank OL712400, IBF20190056D, GenBank OL712402; 22. Aug 2021, leg.: L. Huymann, IBF20210123, GenBank OL712397; Lans, 10. Aug 2010, leg.: L. Huymann, IBF20190002-8; 03. Sep 2019, leg.: L. Huymann, IBF20190002-6; 15. Sep 2019, leg.: L. Huymann, IBF20190002-11; 08. Sep 2020, leg.: L. Huymann, IBF20200034, GenBank MW880276, IBF20200035, GenBank MW880277; 01. Sep 2021, leg.: L. Huymann, IBF20210155; 04. Sep 2021, leg.: L. Huymann, IBF20210167; Mutters, 05. Sep 2019, leg.: L. Huymann, IBF20190002-10; 31. Aug 2020, leg.: L. Huymann, IBF20200032, GenBank MW880274; 08. Sep 2020, leg.: L. Huymann, IBF20200057; 14. Sep 2020, leg.: L. Huymann, IBF20200036, GenBank MW880278; 10. Aug 2021, leg.: L. Huymann, IBF20210112, GenBank OL712391; Natters, 22. Aug 2019, leg.: L. Huymann, IBF20190002-3, IBF20190002-4, IBF20190002-1, IBF20190002-2; 28. Aug 2019, leg.: L. Huymann, IBF20190002-5, IBF20190002-9; Aug 2019, leg.: L. Huymann, IBF20190002-5, IBF20190002-9; Patsch, 20. Sep 2018, leg.: U. Peintnter, IBF20180120, GenBank MW880279; **Italy**: Emilia-Romangna: Abetone, 15. Oct 2018, leg.: L. Huymann, U. Peintner, IBF20180232, GenBank MW880273; Parma: Bedonia, 23. Oct 2019, leg.: L. Huymann, U. Peintner, IBF20190002-7, GenBank MZ357345; Triento: Prad am Stilfserjoch, 30. Jul 2021, leg.: G. Turrini, IBF20210184, GenBank OQ549977.

*Notes*: Phylogenetic analyses clearly show that *C*. *malicorius* and *C*. *rubrophyllus* are sister-taxa. *C*. *rubrophyllus* sequences differ from *C. malicorius* sequences by 8 to 10 bases in the ITS-region. Morphologically, *C*. *malicorius* differs by brighter red orange veil remnants and more brightly orange coloured lamellae, while the lamellae of *C*. *rubrophyllus* are more red*.* Pigment-profiles at 428 nm differ only slightly: *C*. *malicorius* has a small amount of pigment **9**, which is absent in *C*. *rubrophyllus*. Other pigments differ mainly by their concentrations (Fig. 3).

### The Cortinarius ominosus clade

The fruiting bodies of species from the C. semisanguineus clade do have a brownish pileus, a white to yellow brownish stipe but do have bright red lamellae. Taxa can be differentiated based on the pale (not red) colour of the context, a brown universal veil, and the ecology. Spores range from 6.8–8.2 µm in length and 3.7–4.7 in width, with Q values around 1.82.

***Cortinarius ominosus* Bidaud.** Atlas des Cortinaires 6: 190 (1994). [MB#446967] (Fig. 6a, b).

*Synonyms*: *C*. *phoeniceus* var. *semisanguineus* ss. Quelet in Mycological Flora and in Grevilles, *C*. *semisanguineus* ss. Bresadola in Myc.: 646.

*Type*: **France**, Creuse Lac Lavaud; on litter of needles, forest of *Abies alba* with some *Picea abies*, in granite terrain; alt.: 700 m; 19 Oct. 1993; leg. et det.: A. Bidaud; holotype no. 3626 in herb. GK, G435756; Genbank PP001388.

*Description*: *Pileus* 30–60 mm; conical spread with a neat protruding papilla, sharply folded margin; pileus surface hygrophane, fibrillose to scaly-felty; margin is of a deep dark brown due to appressed veil remnants; red–purple-garnet, later mahogany red, copper-brown when drying. *Lamellae* 5–8 mm wide; blood-red; narrowly sinuous-emarginated; edges are crenate and orange-yellow. *Stipe* 40–70 mm × 5–8 mm; subequal or base slightly widened, a little tortuous, hollow, lemon yellow with salmon pink basal mycelium. *Universal veil* forming incomplete bracelets of grey-brown veil starting from the base, sometimes in bands. *Context* red-brown in the pileus, orange-yellow in the stipe and yellowish-brown at the base. *Odour* raphanoid when cut, later cedar wood. *Macrochemical reactions* with KOH black-purple on pileus; garnet in context. *UV*-fluorescence under 350 nm, on stipe (orange) and stipe base (stronger orange). *Basidiospores* (6.84) 7.47 ± 0.34 (8.24) µm x (3.7) 4.12 ± 0.25 (4.7) µm; Q = 1.82 ± 0.12 (n = 31); ovoid; finely ornamented. *Basidia* with four sterigmata, 25–30 µm × 7–10 µm. *Cheilocystidia* in bunches on the edge, formed by short articles; septs 6–9 µm in size. *Pileipellis* with suprapellis formed by large hyphae, 6–12 µm in diameter, with short segments and slightly raised free ends; with parietal pigment encrusting-zebra yellow–brown and carmine-red vascular pigment in some hyphae. *Subpellis* with subcellular tendency 20–25 µm in diameter; with carmine red vascular pigment which is quite concentrated in this sector. Presence of carmine-red interhyphic pigment masses in potash.

*Pigments*: dermolutein-6-glycoside (**4**), emodin-1-glycoside (**6**), dermocybin-1-glycoside (**7**), dermolutein (**8**), dermorubin (**9**), dermocybin (**13**) (traces), and an unidentified pigment around the retention time 5.5.

*Habitat:* montane forests with *Picea abies* and *Betula*.

*Distribution*: Very common in Austria, known from, Finland, France, and Italy.

*Specimens examined*: **Austria**: Tirol: Bogner Aste, 01. Oct 2020, leg.: L. Huymann, IBF20200020, GenBank MW880269; 13. Oct 2020, leg.: L. Huymann, IBF20200021, GenBank MW880270; Lans, 14. Sep 2019, leg.: L. Huymann, IBF20190005-2, IBF20190005-3; 08. Sep 2020, leg.: L. Huymann, IBF20200015, GenBank MW880264, IBF20200016, GenBank MW880265; 21. Sep 2020, leg.: L. Huymann, IBF20200018, GenBank MW880267, IBF20200019, GenBank MW880268; 01. Sep 2021, leg.: L. Huymann, IBF20210154; 03. Sep 2021, leg.: L. Huymann, IBF20210163; Mutters, 10. Aug 2019, leg.: L. Huymann, IBF20190005-1; 14. Sep 2020, leg.: L. Huymann, IBF20200017, GenBank MW880266; 04. Sep 2021, leg.: L. Huymann, IBF20210170; Natters, 24. Sep 2019, leg.: L. Huymann, IBF20190005-4; **Italy**: Triento: Falzes-Pfalzen-Unterberg, 02. Nov 2011, leg.: G. Turrini, IBF20110187, GenBank OQ549964; Falzes-Pfalznerwald, 16. Sep 2022, leg.: G. Turrini, IBF20220176, GenBank OQ549980;, 03. Oct 2022, leg.: G. Turrini, IBF20220177, GenBank OQ549981.

*Notes*: *C*. *ominosus* was earlier annotated as *C*. *semisanguineus* sensu Quélet and Bresadola, but then recognized as a distinct species (Bidaud et al. 1994). We confirm that *C*. *semisanguineus* and *C*. *ominosus* are distinct, although morphologically very similar. *C*. *ominosus* differs from *C*. *semisanguineus* by reddish-brown pilei, brownish veil, and the lack of a red tomentum covering the base of the stipe (as typical for *C. semisanguineus*). The real *C*. *semisanguineus* appears to be a very rare species in Central European coniferous forests. It was detected in a *Picea abies* forest on calcareous bedrock in Southtyrol (Italy, Genbank U56065), and might be restricted to calcareous habitats.

### The Cortinarius sanguineus clade

Fruiting bodies are entirely red, blood red or purple red, or with a brown pileus. A distinct universal veil is missing. Basidiospores are comparatively small with a range from 5.6–7.3 µm in length, 3.1–4.6 µm in width, with Q values around 1.68.

***Cortinarius sanguineus* (Wulfen) Gray.** A natural arrangement of British plants 1: 629 (1821). [MB#177547] (Fig. 6g and h).

*Basionym*: *Agaricus sanguineus* Wulfen, Miscellanea austriaca ad botanicum, chemiam et historiam naturalem spectantia 2: 107 (1781) [MB#241572].

*Type*: **Sweden**, Småland, Femsjö parish, just N of Knapabo; 22. Sep 1940; leg. et. det.: Seth Lundell; neotype UPS SL22091940, designated by Høiland (1983); GenBank JN114099.

*Description*: *Pileus* 25–45 mm; umbonate to plane; pileus surface often hygrophan; rich, bright, to dull red. *Lamellae* bright carmine-red. *Stipe* 30–85 mm × 3–7 mm, red and covered by an orange yellow basal mycelium, mostly on the base. *Context* dark red to red with orange tints towards the base. *Universal veil* dark red, fibrillose. *Taste*. *Odour* cedar tree-like in lamellae. *Macrochemical reaction* with KOH distinctly aniline red in pileipellis. *UV*-fluorescence very weak, slightly red under 350 nm. *Basidiospores* (5.6) 6.3 ± 0.33 (7.3) µm x (3.1) 3.7 ± 0.26 (4.6) µm; Q = 1.68 ± 0.13 (n = 30); amygdaloid to ellipsoid; weakly dextrinoid; moderately verrucose.

*Pigments*: emodin-1,6-di-glycoside (**1**), emodin-1-glycoside (**6**), dermocybin-1-glycoside (**7**), dermolutein (**8**) (traces), dermorubin (**9**) (traces), emodin (**12**), dermocybin (**13**) (traces).

*Habitat*: In mesic to damp mossy coniferous forests with *Picea abies,* rarely with deciduous trees. Specially on nutrient-rich ground. Often found among *Sphagnum* and blueberry spruce in dense forests.

*Distribution*: Common in hemi boreal to boreal climate zones. Known from northern Europe, and montane areas of central and southern Europe (Austria, Denmark, Finland, Norway and Sweden).

*Specimens examined*: **Austria**: Tirol: Bogner Aste, 01. Oct 2020, leg.: L. Huymann, IBF20200046, GenBank MW880289; 22. Aug 2021, leg.: L. Huymann, IBF20210120, GenBank OL712409; Lans, 10. Aug 2010, leg.: L. Huymann, IBF20190006-5; 10. Aug 2019, leg.: L. Huymann, IBF20190006-1; 20. Aug 2019, leg.: L. Huymann, IBF20190006-2; 21. Sep 2020, leg.: L. Huymann, IBF20200043, GenBank MW880286, IBF20200044, GenBank MW880287; 23. Sep 2020, leg.: L. Huymann, IBF20200039, GenBank MW880282; 01. Sep 2021, leg.: L. Huymann, IBF20210146, IBF20210147, IBF20210148, IBF20210149, IBF20210150, IBF20210151, IBF20210152; 03. Sep 2021, leg.: IBF20210164, GenBank OL712406; Mutters, 31. Aug 2020, leg.: L. Huymann, IBF20200037, GenBank MW880280; 14. Sep 2020, leg.: L. Huymann, IBF20200041, GenBank MW880284, IBF20200042, GenBank MW880285; 28. Sep 2020, leg.: L. Huymann, IBF20200040, GenBank MW880283; 02. Oct 2020, leg.: L. Huymann, IBF20200038, GenBank MW880281; Tirol, Mutters, 04. Sep 2021, leg.: IBF20210169, GenBank OL712408; 04. Sep 2021, leg.: IBF20210168; Tirol, Mutters, 08. Sep 2021, leg.: IBF20200063; Natters, 28. Aug 2019, leg.: L. Huymann, IBF20190006-3; 19. Sep 2019, leg.: L. Huymann, IBF20190006-4; 26. Sep 2020, leg.: L. Huymann, IBF20200045, GenBank MW880288; 02. Oct 2020, leg.: L. Huymann, IBF20200072, GenBank OL712403; **Italy**: Triento: Percha, 29. Aug 2006, leg.: G. Turrini, IBF20060553, GenBank OQ549956; **Sweden:** Com. Borgsjoe-Ensillre, Granboda, 24. Aug 2010, leg.: G. Turrini, IBF20100143, GenBank OQ549961.

*Notes*: *C. sanguineus* is easily distinguishable from other species in Europe because of its red coloration. (see *C. vitiosus* for more notes).

### Species without clear affiliation within the Dermocybe clade

***Cortinarius cistoadelphus* (G. Moreno, Pöder, Kirchm., Esteve-Rav. & Heykoop) Huymann & Peintner, comb. nov.** [MB#847170].

Basionym: *Dermocybe cistoadelpha* G. Moreno, Pöder, Kirchm., Esteve-Rav. & Heykoop Mycotaxon 62: 240 (1997) [MB#437089].

*Typus*: **Spain**, Pto. Canamero, Villuercas, beneath *Cistus ladanifer*, acidic ground; 22 Sep. 1994; leg.: V. Gonzáles, G. Moreno, det.: G. Moreno; holotype AH 18359, isotype IBF19940606, GenBank OL712389.

*Description*: *Pileus* 20–55 mm; campanulate to convex or plano-convex, central conical or obtuse umbo; reddish purple. *Lamellae* are blood red. *Stipe* 30–60 mm × 3–8 mm; reddish purple and yellowish on the upper half. *Universal veil* deep red. *Context* wine-purple under the cuticle, yellowish or yellowish-white in the stipe. *Taste* sour. *Odour* not distinct. *Macrochemical reaction* not available. *UV- fluorescens* on stipe dark red to very dark red. *Basidiospores* (6.0) 7.5 ± 0.82 (9.2) µm x (3.7) 5 ± 0.52 (6.0) µm; Q = 1.52 ± 0.21 (n = 38); broadly elliptical and sub-amygdaliform; slightly verucculose.

*Pigments*: dermolutein-6-glycoside (**4**) (traces), emodin-1-glycoside (**6**), dermocybin-1-glycoside (**7**), dermolutein (**8**), dermocybin (**13**).

*Habitat:* with *Cistus ladanifer* often on acid, sandy soil in humus.

*Distribution*: Spain.

Notes: *Cortinarius cistoadelphus* appears to be a rare species which is restricted to Mediterranean *Cistus* habitats. It has sistergroup relationships to *C. purpureus*, which are also reflected by the same pigment type and similar morphology.

***Cortinarius fervidus* P.D. Orton.** Notes from the Royal Botanical Garden Edinburgh 26 (1): 47 (1964). (Fig. 6d).

*Basidionym*: *Cortinarius fervidus* P.D. Orton, Notes from the Royal Botanical Garden Edinburgh 26 (1): 47 (1964).

*Synonym*: *Dermocybe fervida* (P.D. Orton) Tartarat, Bull. trimest. Féd. Mycol. Dauphiné-Savoie 29 (no. 113): 29 (1989).

*Type*: **United Kingdom**, Scottland, Inverness-shire, Loch an Eilean 3; 2. Sep 1960; leg. et. det.: P. D. Orton; holotype E00433637, Orton No. 2222, K109576; GenBank PP001386.

*Description Pileus* 15–80 mm; plane to conical, sometimes the margin tends to rise; pileus surface with a very low-grained glossy coating in the centre; in the middle, the colour is dark purple to almost black, the margin more of a bright orange-red, mainly caused by small radial cracks. *Lamellae* adnate; of a bright orange-red with darker edges, young more deep orange red brown to rusty red. *Stipe* 40–50 mm × 3–14 mm; mostly straight, but gradually bulging towards the base; purple black and resembling the pileus, but with a saffron-yellow to orange-yellow mycelial felt at the base that becomes fawn with age and at the tip more yellow-fawn-olivaceous. *Universal veil* orange, red or red-brown; remnants covering the stipe. *Context* saffron yellow, clearly washed with olive; in stipe orange to rose red. *Odour* radish. *Taste* not available. *Macrochemical reactions* with KOH lamellae and flesh black, cortex of stipe apex dark red. *UV*-fluorescence of the stipe apex (slightly orange) (Bidaud et al. 1994). *Basidiospores*: (4.4) 5.5 ± 0.53 (6.9) µm x (3.3) 3.9 ± 0.35 (4.9) µm; Q = 1.43 ± 0.14; ovoid to amygdaloid; fine and sparsely dense ornamentation. *Basidia* regular; 20–26 µm × 5–7 µm spaced apart. *Pileipellis* with large hyphae (× 5–12 µm); with free, numerous, and sometimes strongly straightened ends. *Subpellis* undifferentiated formed of large oval particles (× 15–20 µm); bright yellow vacuole pigment, becoming rose red in KOH, very concentrated in the first layers; presence of large interhyphic yellow masses in flesh and edges of lamellae; carmine red exsiccates in 5% KOH. *Marginal hairs* clavate or fusiform; articulated (× 5–6 µm).

*Pigments*: emodin-1,6-di-glycoside (**1**), dermolutein-6-glycoside (**4**) (traces), emodin-1-glycoside (**6**), dermocybin-1-glycoside (**7**), dermolutein (**8**), FDM (**11**), emodin (**12**), 7,7’-biphyscion (**15**).

*Habitat*: on intermediate to rich soil, often in damp *Picea* forests, rarely under *Pinus.* Occasional in hemi boreal and alpine, rare in temperate.

*Distribution*: In Europe (known from Austria, Denmark, Finland, Germany, Norway, Sweden, and the United Kingdom).

*Specimens examined*: **Austria:** Tirol: Mutters, 14. Sep 2020, leg.: L. Huymann, IBF20200052, GenBank MW880272; **Germany:** Baden Württemberg, Ehingen, 28. Sep 2010, leg.: G. Turrini, IBF20100144, GenBank OQ549962.

*Notes*: *Cortinarius fervidus* is comparatively rare in Central European coniferous forests. Our collection shares 100% identity with the type sequence. Phylogenetically, it has a sistergroup relationship to *C. rubrobrunneus* (Niskanen 2014) and it appears to be somehow related to *C. pellstonianus*.

***Cortinarius pellstonianus* Ammirati & A.H. Sm.** The Michigan Botanist 11: 13 (1972) (Fig. 6c).

*Synonym*: *Dermocybe pellstoniana* (Ammirati & A.H. Sm.) Ammirati, Mycotaxon 33: 440 (1988). [MB#134675];

*Basionym: Cortinarius sommerfeltii* Høil., Opera Botanica: 97 (1984). [MB#107877].

*Type*: **USA**, Michigan, Emmet Co., Pellston, gregarious under *Populus* (Aspen); 30 Sep. 1962; leg. et det.: A.H. Smith 66395; holotype MICH10394.

*Description*: *Pileus* 13–70 mm; conical to convex; dull ochraceous brown to chestnut brown when young, often with darker, concentrically zones getting bigger when older. *Lamellae* ochraceous orange when young, later more brownish. *Stipe* 38–80 mm × 5–9 mm; pale ochraceous to pale ochraceous yellow, at base usually ochraceous yellow felty. *Context* yellowish, darker at the base, when very old getting a bit reddish. *Universal veil* pale red-brown, ochraceous brown or dirty brown. *Taste* and *Odour* slightly pungent (not truly raphnaoid). *Macrochemical reaction* with KOH rusty brown on pileus. No *UV*-fluorescence. *Basidiospores:* (7.1) 8.2 ± 0.51 (9.3) x (4.5) 5.3 ± 0.33 (6.1) µm; Q = 1.55 ± 0.14; ovoid to amygdaloid; verruculose.

*Pigments*: emodin-1-glycoside (**6**), dermocybin-1-glycoside (**7**) (traces), dermolutein (**8**), dermorubin (**9**), FDM (**11**), emodin (**12**), AFDM (**14**), 7,7’-biphyscion (**15**) (traces).

*Habitat:* associated with *Populus tremuloides*, *Picea* and seldom *Pinus*, in damp, shady coniferous forests. *C. pellstonianus* has circumpolar distribution. It is common in hemi boreal to boreal, and seldom in temperate climate zones.

*Distribution:* In Europe (known from Austria, Denmark, Finland, Italy, Norway, and Sweden).

*Specimens examined*: **Austria**: Salzburg: Innerschwand, 22 Jul 2021, leg.: L. Huymann, U. Peintner, IBF20210093, GenBank OL712390; Tirol: Ellbögen, 22 Aug 2021, leg.: U. Peintner, IBF20210121, GenBank OL712395; Lienz—Iselsberg, 28 Jul 2011, leg.: G. Turrini, IBF20110186, GenBank OQ549963.

*Note*s: Sequences generated from our material (IBF20210093, IBF20210121) are identical to the sequence generated from the type of *C. sommerfeltii* (holotype: OF72584). However, C. *sommerfeltii* Høiland is a later synonym of the American species *C. pellstonianus*. The ITS sequences of the two differ by only one base, and they are morphologically identical.

***Cortinarius purpureus* (Bull.) Bidaud, Moenne-Locc. & Reumaux**. Atlas des Cortinaires. 6:191 (1994). [MB#446973] (Fig. 6f).

*Basionym*: *Agaricus purpureus* Bull. ex Pers., Synopsis methodica fungorum: 290 (1801).

*Synonym*: *Cortinarius phoeniceus* (Bull.) R. Maire, Bulletin Trimestriel de la Société Mycologique de France 27: 434 (1911) [MB#296567].

*Type*: **Sweden**; dry, sandy pine forest; leg. et det.: H. Lindstrom; epitype, CFP742, GenBank NR157868.

*Description*: *Pileus* 15–80 mm; first semiglobose to conical later convex, plane or umbonate; smooth to felty; young red brown, later dark orange red brown to bright red-brown with a blood-red tint, also more dark red carmine with a brown tint, with a lighter margin usually yellow-brownish with red veil remnants. *Lamellae* adnex to adnate; deep blood-red to carmine, later with a chestnut brown tinge. *Stipe* 30–80 mm × 5–12 mm; cylindrical to slightly subclavate; ochraceous yellow or ochraceous orange to pale red-brown, carmine with bright red to orange veil zones. *Basal mycelium* rose to carmine mycelial felt. *Context* in pileus rose to carmine; in stipe pale ochraceous, rose to carmine. *Macrochemical reaction* with KOH pileus context brownish; pileipellis blackish carmine; stipe context red-brown or olivaceous; stipe cortex blackish to carmine*. Odour* in context slightly iodiform. *Taste* not observed. *UV*-fluorescence context and stipe apex very light orange at 350 nm. *Basidiospores* (5.7) 6.6 ± 0.42 (7.2) µm x (3.6) 4.2 ± 0.39 (5.1) µm; Q = 1.56 ± 0.13 (n = 30); usually amygdaloid; finely verrucose (Høiland 1985).

*Pigments*: emodin-1-glycoside (**6**), dermocybin-1-glycoside (**7**), dermolutein (**8**), dermorubin (**9**), dermocybin (**13**) (traces).

*Habitat*: Often on sandy soil with *Pinus*, or on mossy soil in *Picea* forests, seldom with other trees. It grows occasionally in temperate to boreal climate zones.

*Distribution*: In Europe (known from Austria, Denmark, Finland, Italy, Norway, and Sweden).

*Specimens examined:* **Austria**: Tirol: Mutters, 24 Aug 2020, leg.: L. Huymann, IBF20200062; 28 Sep 2020, leg.: L. Huymann, IBF20200053, GenBank MW880271; 10 Aug 2021, leg.: L. Huymann, IBF20210115, GenBank OL712394; **Italy**: Emilia-Romangna: Albareto, 16 Oct 2022, leg.: G. Turrini, IBF20220179, GenBank OQ549983; Trentino: Falzes-Pfalzen, 23 Oct 2022, leg.: G. Turrini, IBF20220180, GenBank OQ549984.

*Notes*: *C*. *purpureus* can be easily distinguished from other red species (e.g. *C*. *vitiosus*) due to its more robust shape, the red-brown pileus, and its pale yellowish stipe with blood red veil girdles (Niskanen et al. 2012).

***Cortinarius vitiosus* (M.M. Moser) Niskanen, Kytöv., Liimat. & S. Laine. **Mycologia. 104(1): 249 (2011). [MB#560217].

*Basionym*: *Dermocybe sanguinea* var. *vitiosa* M.M. Moser, Schweizerische Zeitschrift für Pilzkunde 54: 149 (1976) [MB#352788].

*Type*: **Sweden**, Småland, Femsjö, way to Pellatorpet (Aborrasjö) mossy coniferous forest; 16. Aug. 1974; leg et det.: M. Moser; holotype IBF19740117, GenBank JN114098.

*Description*: *Pileus* 20–50 mm; hemispherical, soon low convex to almost place, often with a small umbo; surface finely fibrillose-tomentose, often with fine fibrillose scales; dark red brown to red often more with more obvious brownish tint when old; somewhat hygrophynous, with hygrophanous zones. *Lamellae* deep carmine. *Stipe* 40–100 mm × 3–8 mm; cylindrical or slightly clavate; deep carmine. *Universal veil* dark red, fibrillose. *Basal mycelium* pale red, sometimes reddish yellow. *Context* in stipe pinkish white, red in cortex. *Taste* not observed. *Odour* in lamellae iodine-like. *Macrochemical reaction* in pileipellis orange-red to somewhat aniline red with KOH. *UV-fluorescence* not available. *Basidiospores* (5.1) 5.8 ± 0.35 (6.7) x (2.9) 3.5 ± 0.21 (3.8) µm; Q = 1.68 ± 0.11 (n = 30, from our Austrian collection); 5.6–6.8 × 3.5–4.2 µm, av. 6.2–6.6 × 3.8–4.0 µm; Q = 1.45–1.8 (n = 120, Niskanen et al. 2012); amygdaloid to ellipsoid; not dextrinoid to weakly dextrinoid; moderately verrucose (Niskanen et al. 2012).

*Pigments*: dermolutein-6-glycoside (**4**) (traces), emodin-1-glycoside (**6**), dermocybin-1-glycoside (**7**), dermolutein (**8**), dermorubin (**9**) (traces), dermocybin (**13**) (traces), AFDM (**14**) (traces), 7,7’-biphyscion (**15**) (very low traces).

*Ecology:* can grow on rather dry or acidic soil than *C. sanguineus* but also on rather moist, mossy soil, in mesic coniferous forests, associated with *Picea*.

*Distribution*: In Europe (known from Austria, Finland, Germany, Italy, Norway, and Sweden).

*Specimens examined*: **Austria**: Tirol: Lans, 09 Sep 2020, leg.: L. Huymann IBF20200047, GenBank MW880293.

*Notes*: *Cortinarius vitiosus* can easily be identified by the combination of having a pileus with a brownish tint, usually pale red basal mycelium, iodine-like odour in lamellae, and small spores. It has often been misidentified as *C. sanguineus* but this was more due to the lack of knowledge of the existence of *C. vitiosus* than these two species actually being identical. *Cortinarius sanguineus* has a red to dusky-red pileus lacking any brownish tints, a reddish-yellow basal mycelium, cedar tree-like odour in lamellae, and larger spores (av. = 7.4–7.9 µm × 4.7–5.1 µm). Additionally, *C*. *sanguineus* occurs on average in richer soils than *C. vitiosus* although the two species can also co-occur (Niskanen et al. 2012).

## Discussion

The main aim of this work was to carry out a molecular revision of central European dermocyboid *Cortinarius* species, and to define characters useful for delimiting and redefining species. The classifications previously proposed did not reflect the true relationships of the species and there was a taxonomic chaos around classical, widely applied epithets. We wanted to compare morphological and pigment chemical characters to phylogenetic relationships – to see if they are resulting in identical or conflicting taxonomic groupings. We now present the allocation of species into their respective clades, but the infrasubgeneric classification of *Dermocybes* will be defined when the phylogenetic relationships are be better resolved. The resolution of subgeneric relationships was not the subject of this work and would be better addressed on the basis of multi-gene phylogenies.

Species recognition has always been challenging in Cortinarius and is especially difficult in groups with low sequence divergence in the rDNA ITS region (Garnica et al. 2016). Many studies (Frøslev et al. 2005; Kõljalg et al. 2013; Stefani et al. 2014; Soop et al. 2019) demonstrated that combining the ITS region with the protein coding RPB1 region offers the best phylogenetic resolution in the genus *Cortinarius*. However, it is often impossible or very difficult to generate protein coding genes from old voucher material. It is therefore highly advisable to first generate multi-copy rDNA sequences from old types, in order to fix the epithet to a lineage. Fresh voucher material belonging to the same lineage can later be used for an in-depth multigene phylogenetic analysis, if necessary.

We found that the rDNA ITS region shows a good phylogenetic resolution at the species level for the subgenus *Dermocybe*, if sampling size is large enough. However, BLAST searches based on ITS alone have often the drawback of detecting too many closely related dermocyboid Cortinarius species, due to their very high percentage of ITS sequence identity (99%), making it difficult to detect the correct species epithet. Due to low sequence divergence, the taxonomic resolution depends very much on sequence quality and length, and resolution can be obtained only based on alignment-based techniques of phylogenetic reconstruction.

Several *Cortinarius* species cannot be separated in a reliable way based on rDNA ITS sequences, especially as long as sampling size is small. As an example, ITS sequence divergence is around 0.5% in ITS, thus making it difficult to differentiate *C. malicorius* from *C. rubrophyllus*, or *C. tubarius* from *C. sphagnogenus*. We were therefore looking for alternative methods helping to define taxa based on old fungarium specimen, even when DNA is already partly degraded.

Environmental characters are certainly very important drivers for the evolution of these wide-spread ectomycorrhizal fungi. Habitat type, host associations and pH of the soil appear to be very strong drivers for the speciaton of these ectomycorrhizal fungi (Høiland 1983). However, they are often poorly documented, especially for older fungarium specimen. This study focused on Central European coniferous forests as the main habitat type. These forests are all quite similar in terms of the pH, climate, and associated vegetation. However, conifers can be mixed with deciduous trees, and especially in their transitional zones, habitats can often overlap. This explains the presence of species associated with *Salix, Betula, Fagus, Quercus* or *Pinus*, or of alpine species like *Cortinaris polaris* in our dataset. The study area was restricted to one habitat type, which could be one possible explanation for the fact, that several “widespread” or well-known species were rarely or never detected in our study. For example, *C*. *croceus* was found only once, and *C. semisanguineus* was not detected at all.

Unfortunately, micromorphological characters useful for distinguishing *Cortinarius* subgenus *Dermocybe* species are mainly limited to basidiospore characters. Basidiospore size is easy to measure, even from old herbarium material, and also spore ornamentation remains unaffected by age, making it a practical tool. Basidiospore size is very useful for distinguishing dermocyboid species groups or clades, but basidiospore size ranges of closely related species do often widely overlap: e.g. *C. malicorius* and *C. rubrophyllus*. We would like to emphasize that, in any case, it is crucial to measure a statistically relevant number (n > 30) of mature basidiospores from the veil or stipe surface of fully developed fruiting bodies. Basidiospore size and ornamentation change during maturation (Høiland 1983), so measuring only a few spores very likely results in incorrect size ranges. But there are other factors as well influencing obtained spore size, e.g. how the spores were measured, which equipment was used and difference in measurements can also be depending on the person taking it. Small differences in spore size should therefore not be over-emphasized and considered only in combination with other characters.

Macromorphology-based identification is still a very useful and fast method for assigning a *Dermocybe* collection to a species complex at least. However, it is often hindered by characters not reported in the original species descriptions: Context colour, odour or KOH reactions are often not reported, although they proved to be very useful differentiating characters. Good and reliable pictures showing the fruitbody context are also very rare for old specimen. Macromorphology-based identification is also difficult based on fresh material: Different collections belonging to one species normally have quite constant and distinct morphological characters, given that the collection includes young and mature fruitbodies. Recognizing fine details (e.g. context colour, discolourations at the base of the stipe) can often be essential for accurately differentiating these fungi. Observing and understanding these often-intricate colour patterns, while seemingly simple, can be challenging and requires a well-trained eye (Moser 1976). One additional character which helps to differentiate closely related *Cortinarius* taxa might be UV-fluorescence (Ammirati et al. 2021). We suggest to generally include this simple method in future in new descriptions of *Cortinarius* spp. However, there are still many closely related species which are tricky to differentiate morphologically, even for the trained Cortinariologist. Therefore, it is often essential to combine morphological character traits with the most reliable identification method, which is still ITS sequencing due to the extensive sequence databases available (UNITE, NCBI).

Taxonomic resolution can be significantly improved, especially when a combination of independent characters is used for species definition. We were especially interested in the resolution of the pigment character sets and whether or not they were resulting in identical or conflicting taxonomic groupings when compared to phylogeny. This is not a new idea, as Moser (1985) tested the potential of additional characteristics gaining a better and more reliable resolution. Nowadays we have the possibility to test characters in comparison to homologous characters, e.g. multigene phylogenies or phylogenomics.

Chemotaxonomy has long been considered the key to solving the puzzling species definitions of these colourful fungi. However, pigment profiles of *Cortinarius* have often been approached by thin layer chromatography alone. We would like to point out that only a correct annotation of the pigments can provide a solid pigment database for *Cortinarius*, which then can be further analysed. One potential application of such data is the understanding of the evolutionary history of pigment synthesis pathways. Our study now indicates that pigment evolution is very likely polyphyletic, as similar or identical pigments were detected in different evolutionary lineages of dermocyboid Cortinarii. However, this hypothesis will be investigated in depth based on a global sampling of dermocyboid Cortinarii. A pigment database is also a prerequisite allowing to identify taxa based on pigment profiles. Modern chemical analysis methods as applied in this work, have now shown, that pigment profiles of dermocyboid *Cortinarius* species are indeed useful and valid characters for species delimitation, especially when pigment concentrations are considered in addition to pigment composition (Hannecker et al. 2023). Pigments or fingerprints of other metabolites, e.g., of volatile organic carbons, have also proven to be suitable tools for species recognition in fungi (Peintner et al. 2019; Telagathoti et al. 2021). While the evaluation of pigment profiles is not yet extensive enough to offer a broad database, the possibility of pigmentomics should be considered in the future. Combining molecular biology and metabolomics or pigmentomics could be a promising and exciting field for mushroom identification and taxonomy. Once the taxonomy is resolved, and reliable reference databases are established for both, molecular data and pigment profiles, a machine learning software could be introduced as a useful, reliable and very fast identification tool of dermocyboid Cortinarii from pigment profiles. Due to the quite consistent results in pigment profiles, a software linked with HPLC–MS and a reliable database is certainly providing good resolution on species level (Zuffa et al., 2024). For the future, we are extending the database with fungal pigment profiles. Databases, like set up by Zuffa et al. (2024) are depending on a prior, correct annotation of the species. DNA-based annotation of species can be difficult in a more interdisciplinary environment (chemistry, pharmacy) due to lack of knowledge or equipment. Through this work, species identification through pigments is possible within the dermocyboid Cortinarii, this could be a great enrichment for applied sciences.

Pigment profiles are highly reproducible within collections, and between different collections belonging to the same species. However, different parts of the fruitbody (e.g., pileus, lamellae, stipe) can have different concentrations and compositions of pigments (Siewert et al. 2022). Therefore, it is advisable to use more than one fruiting body or, at least, a whole fruiting body for pigment analysis. Unfortunately, it is often difficult to obtain such large amounts of material from precious herbarium material such as type material. But here the question arises whether or not pigment characters remain constant over time. We compared pigment profiles of recent *C. holoxanthus* collections with profiles from its 58 years old type material and found that some pigments, such as FDM (**11**) and 7,7’-biphyscion (**15**), changed their structure over time: FDM content was reduced, 7,7’-biphyscion increased due to oxidation. Pigment profiles can reliably be measured even after at least 40–60 years of preservation as dried fruiting bodies. This highlights that it is permissible to use herbarium material for species delimitation through chemotaxonomical traits. We thus confirm that, as earlier suggested (Kidd et al. 1985), it is possible to (re-)identify old herbarium material based on their pigment profiles. However, due to structural changes, the results have to be carefully considered, and should be complemented with pigment data from fresh collections (Fig. 2).

Pigment patterns have long been used to distinguish *Cortinarius* species: Already Keller (1982) grouped *Cortinarius* subgenus *Dermocybe* species, which produce similar pigment patterns into five higher ranking pigment types (Keller 1982). Now we know that these pigment types or groups are not consistent with taxonomical groups (lineages). Pigment groups should now help to understand the distribution of pigments in the genus, and help to evaluate the potential function of these substances in nature, and their potential as pharmaceuticals. Kellers groups where: The anthracina-pigmentation type, the cinnabarina-pigmentation-type, the cinnamomea-pigmentation-type, the malicoria-pigmentation-type, and the sanguinea-pigmentation-type. *Cortinarius anthracinus,* the only member of the anthracina-pigment-type, and *C. cinnabarinus* representing the cinnabarina-pigmentation type is no longer considered as members of the subgenus *Dermocybe*. Keller (1982) assigned the following species to the cinnamomea pigmentation-Type: *C*. *alnophilus*, *C*. *carpineti*, *C*. *cinnamomeus*, *C*. *cinnamomeoluteus*, *C*. *croceus*, *C*. *holoxanthus*, *C*. *sphagneti*, and *C*. *uliginosus*. We have revised this pigment type and renamed it Croceus-pigment group, as *C*. *cinnamomeus* is not assigned to this pigment group due to the presence emodin-1-glycoside (**6**), among other differences. According to our results, the following species are included in the Croceus-pigment type: *C*. *hadrocroceus*, *C*. *holoxanthus*, *C*. *huronensis*, and *C*. *salignus*. We propose to include *Cortinarius cinnamomeus* in the Malicorius-pigment group, together with *C*. *malicorius*, *C*. *rubrophyllus*, *C*. *pellstonianus*, and *C. fervidus* (Fig. 3). This group stands out by the presence of pigments of the endocrocin-, emodin- and flavomannin type (Keller 1982). *C. cinnamomeus* is only tentatively included here due to its comparatively low concentrations of FDM (**11**). We found that pigment-groups do generally have fluent transitions. Some species cannot easily be assigned to any group. For instance, *C*. *pellstonianus* lacks emodin-1.6-diglycoside (**1**), dermolutein-6-glycoside (**4**) and AFDM (**14**), which distinguishes it from the rest of the Malicorius-pigment group. We defined the Ominosus-pigment group for *C*. *ominosus* and *C*. *sphagnogenus*. These taxa lack FDM (**11**), 7.7’-biphyscion (**15**), or AFDM (**14**). Interestingly, they produce a high number of pigments in low concentrations. This especially concerns *C*. *ominosus* and might reflect the micromorphology as its lamellae are red, whilst the pileus and stipe are brownish/yellow. The Sanguineus-pigment group includes *C*. *cistoadelphus*, *C*. *sanguineus, C*. *purpureus*, and *C*. *vitiosus*. We found that especially this pigment group needs careful revision. Pigment data are often not reliable due to the fact that there were, and still are, many wrongly assigned collections of *Dermocybe* in herbaria and databases. The epithet *C. sanguineus* was initially applied for all all-red specimen of the *Dermocybe* group (Liu et al. 1995). Collections earlier identified as *C. sanguineus* are now known to include several species of which many are not even closely related phylogenetically: *C. cistoadelphus*, *C. marylandensis, C. purpureus*, *C. sierraensis,* and *C. vitiosus* (Liu et al. 1995, 1997; Moreno et al. 1997; Niskanen et al. 2012, 2013). Pigment profiles need to be assessed for each of these species in order to allow meaningful distinctions based on or including pigment pattern.

Last not least, *Cortinarius* pigments do have a huge biotechnological potential. A better knowledge of the pigment structure and the isolation of individual pigments allow a deeper insight into the possible biological function and application of such metabolites. *Dermocybe* pigments are not only useful and promising substances as dye (Räisänen 2019). More importantly, pigments from *Dermocybe* appear to be a promising source for novel photopharmaceuticals, and could thus help to overcome the current antibiotic crisis (Siewert 2021; Hammerle et al. 2022).

Summarizing above-mentioned consideration, our study confirms that the diversity of *Cortinarius* subgenus *Dermocybe* is generally higher than assumed in montane to subalpine *Picea abies* dominated forests, and that a careful evaluation of characters is necessary for a meaningful species delimitation. In addition to a phylogenetic placement based on ITS sequences, we consider the following characters as essential for species definition: i) host associations of these ectomycorrhizal fungi; ii) fruiting body morphology, which must include context and velum colours, KOH reaction and UV-fluorescence; iii) basidiospore size and ornamentation; iv) pigment pattern. Advances in biostatistics have made it easier to define species based on a fuzzy set of characters, e.g. by a combination of pigment profiles, sequences, and morphological data, but only if databases are available and continuously built up. This could help to identify fungal taxa based on metabolite profiles (Zuffa et al. 2024). A full evaluation of above characters is strongly advised for all taxa newly described in the future.

## Conclusion

The in-depth analysis of dermocyboid *Cortinarius* species found in Central European *Picea abies* forests confirmed our hypothesis that the actual species diversity is much larger than currently estimated. The true diversity is blurred by too wide and wrong species concepts of several old, Friesian species like *C. croceus* and *C. cinnamomeus* or *C. malicorius*. Molecular and chemotaxonomical studies carried out in parallel with careful phenotypical analyses resulted in a good differentiation of species. The rDNA ITS region had—in most cases—good resolution on species level, as long as taxon sampling was sufficient. Pigment analyses provided a good separation of closely related species. Our results confirmed pigment analyses as useful for an accurate taxonomic resolution in this group, as they are a good tool for a more solid distinction of closely related species, and of use for species assignment.

## Key to the species of *Cortinarius* subgenus *Dermocybe* from Central European coniferous forests

This key should be useful for identification of taxa occurring in the European alpine range, in coniferous forests. It is mainly based on macroscopical characters and should thus enable an identification to a broad mycological community. Species with an asterisk* were not confirmed with a type sequence or outside the scope (habitat) of this study.

- 1 Lamellae orange or red when young …………………………………………………………………………………….. 2
- 1* Lamellae are mainly yellow when young ……………………………………………………………………………. 12
- 2 Lamellae are mainly orange when young ………………………………………………………………………………. 3
- 2* Lamellae are somewhat red (rust to carmine red) when young, sometimes dull brownish red ……………………………………………………………………………………………………………………………………………. 7
- 3 Veil brownish, ochraceous or inconspicuous; context pale, lemon yellow to yellow-ochraceous; lamellae bright orange to ochraceous dull orange …………………………………………………………………….. 4
- 3* Veil bright orange; flesh yellow or olive-brown to yellowish green (kind of dirty looking); lamellae bright orange to orange-red …………………………………………………………………………………………………… 5
- 4 Lamellae bright orange when young; pileus young evenly yellow brown (cinnamon) to orange brown, later cinnamon yellow to red brown or chestnut brown; stipe pale (lemon) yellow; spores (4.5) 6.1 ± 0.69 (7.6) µm x (3.2) 4.3 ± 0.56 (5.4) µm; ovoid to amygdaloid; finely to moderate verrucose …… *Cortinarius cinnamomeus* (L.) Gray
- 4* Lamellae ochraceous dull orange when young not vivid but more with a brownish tinge; pileus umber to dull ochraceous to chestnut brown, often with concentrically zones; stipe pale ochraceous, often zonate covered with pale brown veil remnants; whole fruitbody with a dull appearance, often with *Populus tremula*; spores (6.8) 8.2 ± 0.51 (9.3) x (4.5) 5.3 ± 0.33 (6.1) µm ovoid to amygdaloid, verruculose ………………………………………………………… *Cortinarius pellstonianus* Ammirati & A.H. Smith
- 5 Pileus red-brown, yellow brown or hazel brown, with orange tinge on the margin of the pileus through orange velum remains; lamellae pure and lively orange to red–orange ……………………………………………………………………………………………………………………………………………. 6
- 5* Pileus bright red-brown to purplish-chestnut, with orange-red, red-brown veil remnants; lamellae more brick red or reddish orange ……………………………………………………………………………………………. 7
- 6 Context yellow to slightly olive in pileus and stipe; stipe yellow, covered with bright orange veil remnants; lamellae bright orange; spores (5.7) 6.6 ± 0.44 (7.4) µm x (3.3) 3.9 ± 0.35 (4.8) µm, ovoid to amygdaloid, finely ornamented …………………………………………………………. *Cortinarius malicorius* Fries
- 6* Context dirty olivaceous in pileus, in stipe yellow at top, blackish brown at the base of the stipe; orange veil remnants on stipe, stipe base sometimes carmine red on surface; lamellae more dull, orange with reddish tint; spores (5.2) 6.0 ± 0.39 (7.0) µm x (3.3) 3.9 ± 0.35 (4.9) µm, elliptical, finely ornamented …….. *Cortinarius rubrophyllus* (Moënne-Loccoz) Liimateinen., Niskanen, Ammirati & Dima
- 7 Pileus and base of the stipe predominantly with ochraceous to brown colour, stipe also with yellow tones …………………………………………………………………………………………………………………………………… 8
- 7* Pileus and stipe with predominantly red colour: dark red, blood red, dark brownish-red, carmine, pinkish ………………………………………………………………………………………………………………………………. 11
- 8 Pileus darkish red brown, umbra-brown to purplish chestnut; lamellae when young deep orange red brown to carmine red; stipe bright, lemon to brownish yellow, with orange red or brown veil remnants, base sometimes reddish when touched. KOH reaction orange-red on pileus; spores (4.4) 5.5 ± 0.53 (6.9) µm x (3.3) 3.9 ± 0.35 (4.9) µm, ovoid to amygdaloid, fine and sparsely dense ornamentation ………………………………………………………………………………………………….. *Cortinarius fervidus* P.D. Orton
- 8* Pileus brown to orange with paler margin lacking warm red tones; lamellae young dark blood red, red brown or dark carmine ……………………………………………………………………………………………………… 9
- 9 Stipe often broad, ochraceous to lemon yellow, densely covered by distinct red velum girdles; pileus brown brown, to copper brown, KOH reaction anilin-red on pileus; spores (5.7) 6.6 ± 0.42 (7.2) µm x (3.6) 4.2 ± 0.39 (5.1) µm, usually amygdaloid, finely verrucose …………………..
- ……………………………………………. *Cortinarius purpureus* (Bulliard) Bidaud, Moënne-Loccoz & Reumaux
- 9* Stipe surface pale yellow to whitish, veil remnants if present not red …………………………………….. 10
- 10 Stipe surface pale to yellow brownish, stipe base often red especially when touched; pileus reddish-brown; context pale; lamellae dark red to carmine; stipe with orange fluorescence under UV light; spores (6.8) 7.5 ± 0.34 (8.2) µm x (3.7) 4.1 ± 0.25 (4.7) µm, ovoid, finely ornamented ……………………………………………………………………………………………………… *Cortinarius ominosus* Bidaud
- 10* It was never detected – missing or very rare in Central European coniferous forests ……………………………………………………………………………. *Cortinarius semisanguineus* (Fries: Fries) Fries*
- 11 Fruitbody red to deep carmine without brownish tint on pileus; stipe base sometimes with orange-yellow to pinkish hue. spore diam. mostly > 4.5 µm; (5.6) 6.3 ± 0.33 (7.3) µm x (3.1) 3.7 ± 0.26 (4.6) µm; amygdaloid to ellipsoid; weakly dextrinoid; moderately verrucose ………………………………………………………………………………… *Cortinarius sanguineus* (Wulfen: Fries) Fries
- 11* Pileus when young somewhat brownish, later brown–red, dark red to red; fruitbody otherwise red to deep carmine; stipe at base with pinkish mycelium, on top surface with whitish hue; spore diam. mostly < 4.5 µm; (5.1) 5.8 ± 0.35 (6.7) x (2.9) 3.5 ± 0.21 (3.8) µm; amygdaloid to ellipsoid, not dextrinoid to weakly dextrinoid, moderately verrucose …… *Cortinarius vitiosus* (M.M. Moser) Niskanen, Kytövuori, Liimatainen & S. Laine
- 12 Stipe with orange or reddish colours at the base, not from the veil remnants, sometimes only when bruised ………………………………………………………………………………………………………………………………. 13
- 12* Stipe base without bright orange or red tinges …………………………………………………………………… 14
- 13 Fruitbodies with dull olivaceous appearance; stipe more whitish creme yellow sometimes with greyish veil remnants as girdles; stipe base in context and flesh reddish after touch or when bruised; in coniferous forests; spores (7.9) 8.7 ± 0.48 (9.9) µm x (4.1) 5.1 ± 0.36 (6.1) µm, ovoid to elliptical, finely warty ………………………………….. *Cortinarius huronensis* (Ammirati & A.H. Smith) Ammirati & A.H. Smith
- 13* Fruitbodies vividly coloured; stipe base colour vivid yellow, with brownish veil girdles, surface and flesh in lower part orange to intensely red; in coniferous forests, also with *Betula* or *Quercus*; lamellae yellow when young; pileus ochraceous yellowish brown with olivaceous tinge …………………………………………………………………………………. *Cortinarius bataillei* (M.M. Moser) Høiland
- 14 Stipe with grey, brown or reddish veil remnants often in girdles …………………………………………… 15
- 14* Stipe without or with not visible yellow veil remnants ……………………………………………………….. 22
- 15 Pileus brick red, orange red, red brown, copper, russet to dark brown or light brown, but never with greenish olive tinge ……………………………………………………………………………………………………………… 16
- 15* Pileus olive brown to yellow olive, specially towards margin ………………………………………………. 20
- 16 Veil remnants on stipe brown or grey ………………………………………………………………………………… 17
- 16* Veil remnants on stipe brown to brick red; with *Salix;* It was never detected – missing or very rare in Central European coniferous forests …………………………………………… *Cortinarius uliginosus* Berkeley*
- 17 Pileus context in the middle light yellow to white when young; lamellae bright yellow to mustard yellow when young ……………………………………………………………………………………………………………… 18
- 17* Context in pileus dark, yellow brown to olive greyish when young; lamellae not so bright yellow, more greyish yellow; stipe with or without brown veil remnants ……………………………………………………….. *Cortinarius davemallochii* Ammirati, Niskanen & Liimatainen
- 18 Among *Salix herbacea*, *S. polaris* or *S. reticulata*; pileus dark chestnut brown to dark hazel brown with orange red tinge; stipe yellow with brown veil remnants; often in cold areas with higher altitude; ………………………………………………………………………………………………………… *Cortinarius polaris* Høiland
- 18* Not among *Salix*, with *Pinus* …………………………………………………………………………………………….. 19
- 19 Stipe whitish with lots of pale reddish to red-brown veil remnants; pileus first tawny brick red, red brown or orange brown, then darker red brown to chestnut; with *Pinus* or *Betula* …….. ……………………………………………………………………………………………………. *Cortinarius croceoconus* Fries
- 19* Stipe whitish to yellow, with brown remnants; pileus without the strong reddish tinge when young ……………………………………………………………………………. *Cortinarius incognitus* Ammirati & A.H. Smith*
- 20 Veil remnants on stipe brown or grey …………………………………………………………………………………. 21
- 20* Veil remnants on stipe rose to yellow brown; It was never detected –very rare in Central European coniferous forests ……………………………………….. *Cortinarius uliginosus* var. *luteus* Gabriel & Lamoure*
- 21 Stipe with pale to yellow base, veil remnants on stipe grey, sometimes inconspicuous, base can get orange to red when touched; pileus hazel brown to dark yellow brown with olivaceous tinge; lamellae olivaceous yellow when young; spores (7.9) 8.7 ± 0.48 (9.9) µm x (4.1) 5.1 ± 0.36 (6.1) µm, ovoid to elliptical, finely warty ………….. *Cortinarius huronensis* (Ammirati & A.H. Smith) Ammirati & A.H. Smith
- 21* Stipe pale yellow to yellow coloured, brown veil forming incomplete girdles on the stipe; pileus dark brown at the centre, when young pale olivaceous yellow, lighter toward the margin; lamellae young olive yellow, later light olive brown; basal mycelium yellow; spores (6.9) 7.8 ± 0.43 (8.9) µm x (3.9) 4.5 ± 0.32 (5.2) µm, amygdaloid, moderately verrucose ………………………………. *Cortinarius hadrocroceus* Ammirati, Niskanen, Liimatainen & Bojantchev
- 22 With *Salix* ……………………………………………………………………………………………………………………….. 23
- 22* With *Pinus*, *Picea* or *Betula;* not with *Salix* …………………………………………………………………………. 24
- 23 With *Salix;* pileus yellow to yellow-olive, later more olive to brown; stipe base with olive mycelium; It was never detected – missing or very rare in Central European coniferous forests …………………………………………………………………………………. *Cortinarius cinnamomeoluteus* P.D. Orton*
- 23* Pileus olive yellow, later more olive; Lamellae yellow; stipe lively yellow, base with olive mycelium which, later mixes into the yellow a red brownish tinge; context in pileus watery olive yellow, in stipe lively yellow; Lamellae with KOH orange brown, Pileus with KOH dark red brown; with Salix; spores (8) 9.6 ± 0.62 (10.8) µm x (5.2) 6.0 ± 0.45 (6.9) µm, elliptical to subamygdaloid, very finely warty………………………………………………… *Cortinarius salignus* (M.M. Moser & Gerw. Keller) G. Garnier
- 24 Lamellae when young yellow to greyish yellow without green or olive tinge …………………………… 25
- 24* Lamella when young olive greenish yellow ……………………………………………………………………….. 26
- 25 coniferous forests; context in pileus yellow; stipe and stipe context lemon yellow; fruitbodies often delicate, lively lemon-yellow appearance; pileus olivaceous yellow, with fine appressed fibres which become darker (light brownish, olive) when older, especially in the middle of the pileus; spores (7.4) 9.1 ± 0.56 (10.5) µm x (4.3) 5.3 ± 0.44 (6.2) µm, elliptic to subamygdaloid, finely to moderately warty …………… *Cortinarius holoxanthus* (M.M. Moser & I. Gruber) Nezdoiminogo
- 25* coniferous forests or with *Betula*; context in pileus yellow white; pileus green brownish with slightly yellow margin; only little veil remnants, if visible dark; spores (7.5) 8.7 ± 0.58 (10.4), (4.1) 5.2 ± 0.48 (6.7), punctate to verrucose …………………………………… *Cortinarius croceus* (Schaeffer: Fries) Gray
- 26 Other habitats………………………………………………………………………………………………………………… 27
- 26* On swampy ground ………………………………………………………………………………………………………. 28
- 27 With *Pinus*, pileus dark brown, lamellae with KOH brown to red brown *……………………………………………………………………………………………….. Cortinarius chrysolitus* Kauffman
- 27* With *Betula* and *Pinus*, lamellae with KOH always dark black brown; pileus olive-yellow brownish, rusty brown olive brown to dark brown, with stronger olive tinge; stipe is first yellow and later rusty-brown to olivaceous; lamellae very young olive greenish …………………………………………………………………………………. *Cortinarius tubarius* Ammirati & A.H. Smith
- 28 With *Fagus, Quercus, Caprinus* in deciduous forests or mixed with conifers; pileus olivaceous green to olivaceous yellow later olivaceous brown, lastly rather dark to hazel brown; lamellae olivaceous yellow or olivaceous green when young; stipe ground colour pale olivaceous green, the top paler, downwards more grey brown to blackish brown; not hygrophan; with KOH red brown, in context rose; It was never detected – missing or very rare in Central European coniferous forests …………………………………………………………………………………………… *Cortinarius olivaceofuscus* Kühner*
- 28* With *Pinus;* pileus ochre to yellow–brown, also tending to umber brown, middle to cinnamon brown, older specimens pronounced reddish brown; lamellae yellow olive; stipe young yellow, when older more olivaceous; KOH reaction not red, at most red-brownish
- ……………………………………………………………… *Cortinarius sphagnogenus* (M.M. Moser) Nezdoiminogo

## Supplementary Information

Below is the link to the electronic supplementary material.

Supplementary file1 (ZIP 18 KB)

Supplementary file2 (ZIP 2159 KB)

## Data Availability

The ITS sequences of all studied fungi are deposited in GenBank. Please refer to supplementary material for a detailed table containing all numbers. Voucher material of all studies species is deposited in the Tiroler Landesmuseum Ferdinandeum (IBF) in Hall, Austria. Other datasets used during the current study are available from the corresponding author on reasonable request.
