# Supplementary material for: Revised taxon definition in European Cortinarius subgenus Dermocybe based on phylogeny, chemotaxonomy, and morphology
Source: Mycol Prog. 2024 Apr 5;23(1):26. doi: 10.1007/s11557-024-01959-z (PMC10997704; doi:10.1007/s11557-024-01959-z)
Supplement: Supplementary file 2 — Supplementary file2 (ZIP 2159 KB) [file 11557_2024_1959_MOESM2_ESM.zip › Additional_file_2_Figure7.docx]

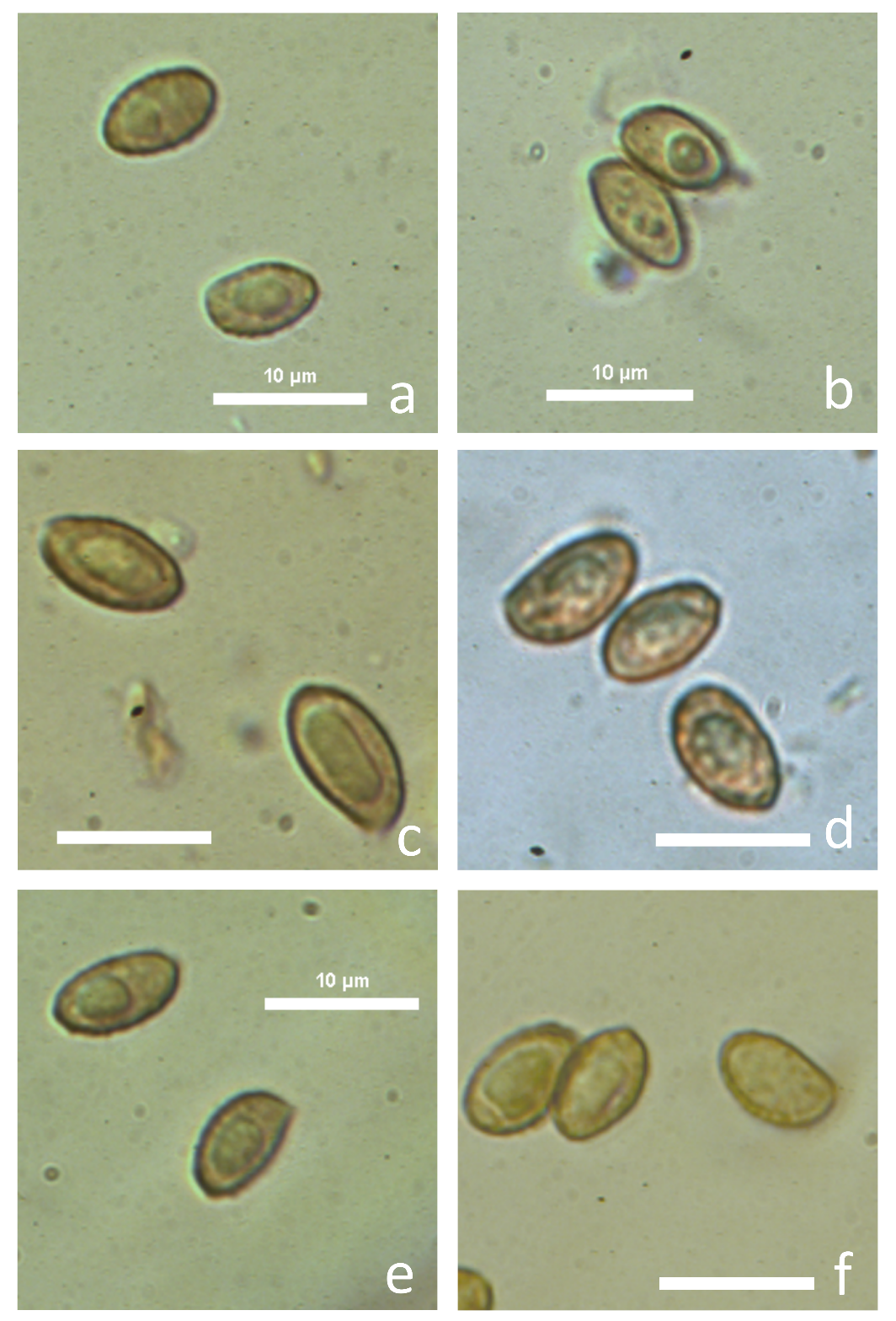


**Fig. 7:** Photographs of basidiospores; a: *C. cinnamomeus* (TG2005234), b: *C. pellstonianus* (TG2011042), c: *C. holoxanthus* (TG2022019), d: *C. croceus* (TG2022099), e: *C. hadrocroceus* (TG2022027), f: *C. huronensis* (TG2018100)
